# Circulating Cardiovascular Proteomic Associations With Genetics and Disease

**DOI:** 10.1161/CIRCGEN.124.005005

**Published:** 2025-10-07

**Authors:** Kathryn A. McGurk, Lara Curran, Arunashis Sau, Fu Siong Ng, Brian Halliday, James S. Ware, Declan P. O’Regan

**Affiliations:** 1 National Heart and Lung Institute, Imperial College London, United Kingdom (K.A.M., L.C., A.S., F.S.N., B.H., J.S.W.); 2 MRC Laboratory of Medical Sciences, Imperial College London, United Kingdom (K.A.M., L.C., J.S.W., D.P.O’R.).; 3 Program in Medical and Population Genetics, The Broad Institute of MIT & Harvard, Cambridge, MA (K.A.M., J.S.W.).; 4 Royal Brompton & Harefield Hospitals, Guy’s & St. Thomas’ NHS Foundation Trust, London, United Kingdom (L.C., B.H., J.S.W.).; 5 Department of Cardiology, Imperial College Healthcare NHS Trust, London, United Kingdom (A.S., F.S.N., J.S.W.).; 6 Chelsea & Westminster Hospital NHS Foundation Trust, London, United Kingdom (F.S.N.).

**Keywords:** angiotensins, diabetes, genetic variation, heart failure, hypertension, proteomics

## Abstract

**BACKGROUND:**

The analysis of the circulating proteome can identify translational modifiers and biomarkers of disease expressivity and severity at a given time point. Here, we explore the relationships between protein measures implicated in cardiovascular disease and whether they mediate causal relationships between cardiovascular risk factors and disease development.

**METHODS:**

To understand the relationships between circulating biomarkers and genetic variants, medications, anthropometric traits, lifestyle factors, imaging-derived measures, and diagnoses of cardiovascular disease, we undertook in-depth analyses of measures of 9 plasma proteins with a priori roles in genetic and structural cardiovascular disease or treatment pathways (ACE2 [angiotensin-converting enzyme 2], ACTA2 [actin alpha 2], ACTN4 [actinin alpha 4], BAG3 [BAG cochaperone 3], BNP [B-type natriuretic peptide], CDKN1A [cyclin-dependent kinase inhibitor 1A], NOTCH1 [neurogenic locus notch homolog protein 1], NT-proBNP [N-terminal pro-B-type natriuretic peptide], and TNNI3 [troponin I]) from the Pharma Proteomics Project of the UK Biobank cohort (over 45 000 participants sampled at recruitment).

**RESULTS:**

We identified significant variability in circulating proteins with age, sex, ancestry, alcohol intake, smoking, and medication intake. Phenome-wide association studies highlighted the range of cardiovascular clinical features with relationships to protein levels. Genome-wide genetic association studies identified variants near *GCKR*, *APOE*, and *SERPINA1*, that modified multiple circulating protein levels (BAG3, CDKN1A, and NOTCH1). NT-proBNP and BNP levels associated with variants in *BAG3*. ACE2 levels were increased with a diagnosis of hypertension or diabetes, particularly in females, and were influenced by variants in genes associated with diabetes (*HNF1A* and *HNF4A*). Two-sample Mendelian randomization identified ACE2 as protective for systolic blood pressure and type-2 diabetes.

**CONCLUSIONS:**

From a panel of circulating proteins, the results from this observational study provide evidence that ACE2 is causally protective for hypertension and diabetes. This suggests that ACE2 treatment may provide additional protection from these cardiovascular diseases. This study provides an improved understanding of the circulating pathways depicting cardiovascular disease dynamics.

Circulating proteomics provides information on the landscape of biological function, metabolism, disease, and homeostasis. Although genetic testing can provide a once-off, invariable assessment from birth, proteome analyses can identify translational modifiers and biomarkers of disease expressivity and severity at a particular sampling time point. In addition to large-scale proteome-wide discovery and risk score analyses, in-depth assessments of selected proteins based on a priori implication in disease are required to fully identify and interpret the relationships between protein measures of interest and cardiovascular disease, and whether they mediate causal relationships between cardiovascular risk factors and disease development.

To understand these relationships in the context of proteins with roles in structural cardiovascular disease, diagnostics, or treatment pathways (aortopathies, cardiomyopathies, congenital heart disease, and heart failure^1^), we undertook an in-depth assessment of the circulating levels of 9 plasma proteins that were measured by the Pharma Proteomics Project of the UK Biobank (UKB) cohort: ACE2 (angiotensin-converting enzyme 2), ACTA2 (actin alpha 2), ACTN4 (actinin alpha 4), BAG3 (BAG cochaperone 3), BNP (B-type natriuretic peptide), CDKN1A (cyclin-dependent kinase inhibitor 1A), NOTCH1 (neurogenic locus notch homolog protein 1), NT-proBNP (N-terminal pro-B-type natriuretic peptide), and TNNI3 (troponin I).

Members of protein families were included when measured in the UKB. This includes actin and actinin-related proteins (ACTA2, ACTN4), proteins with genetic evidence for involvement in structural cardiovascular disease (BAG3, CDKN1A, NOTCH1, TNNI3), and proteins targeted as biomarkers or treatment of disease (ACE2, BNP, NT-proBNP). ACE2, with functionally opposing roles of ACE1 (which is unmeasured here, is the target of ACE inhibitors, and is involved in the biosynthesis of the angiotensin II vasoconstrictor), is a part of the renin angiotensin aldosterone system that regulates blood pressure by catalyzing the hydrolysis of vasoconstrictor angiotensin II and creating other vasodilator angiotensins. ACTA2 is an actin protein involved in the contraction of smooth muscle, and genetic variants in *ACTA2* cause autosomal dominant familial thoracic aortic aneurysm and aortic dissection.^2^ ACTN4 is the only α-actinin measured in a family of actin-binding proteins. ACTN4 has a calcium-binding domain and has been recently identified in vitro in the cardiac Z-disc.^3^ BAG3 is involved in chaperone-assisted selective autophagy of damaged cytoskeletal components, and variants in the *BAG3* gene cause autosomal dominant dilated cardiomyopathy and myofibrillar myopathy.^1,4^ The genetic locus of CDKN1A has been recently identified in case-control genome-wide association study (GWAS) analyses of cardiomyopathies,^5,6^ and it is a regulator of cardiomyocyte cell cycle arrest.^7^ NOTCH1 controls cell fate decisions and variants at the locus have been previously associated with congenital heart disease^8^ and trabeculation.^9^ Prepro-BNP and pro-BNP (both unmeasured here) are cleaved by a convertase to create BNP and NT-proBNP (inactive with a longer half-life than BNP); both are clinical biomarkers upregulated with heart failure and myocardial stretching. Prepro-BNP is encoded by *NPPB* and has unique expression in the heart, highest in the atrial appendage (Genotype-Tissue Expression project).^10^ BNP has roles in natriuresis, diuresis, and vasodilatation. TNNI3 is a mediator of relaxation in the sarcomeric thin filament of cardiac striated muscle and is exclusively expressed in the heart. Variants in *TNNI3* cause autosomal dominant hypertrophic cardiomyopathy.^1,11^

Exploration of the relationships between circulating proteins and upstream factors that may influence their levels (eg, anthropometric traits and protein quantitative trait loci identifiable from genome-wide common and rare genetic factors) as well as downstream clinical end points they may predict or prevent (cardiovascular diagnoses, clinical features, and ECG and MRI-derived traits), would aid our understanding of these biomarkers of cardiovascular disease. Here, we use the results of the Pharma Proteomics Project of the UKB cohort: Olink proteomic data from plasma samples collected at recruitment. We analyzed the proteomics from over 45 000 participants to identify relationships with the full spectrum of available UKB data and tested for causal relationships.

## Methods

### Study Overview

All data have been made publicly available through the UKB (https://biobank.ndph.ox.ac.uk/showcase/) and the GWAS catalog (https://www.ebi.ac.uk/gwas/). The UKB cohort study recruited 500 000 participants aged 40 to 69 years old from across the United Kingdom between 2006 and 2010.^12^ The study received ethical approval from the National Research Ethics Service (11/NW/0382), and all participants gave written informed consent. This research has been conducted under application numbers 47602 and 40616.^12^

**Figure 1.**
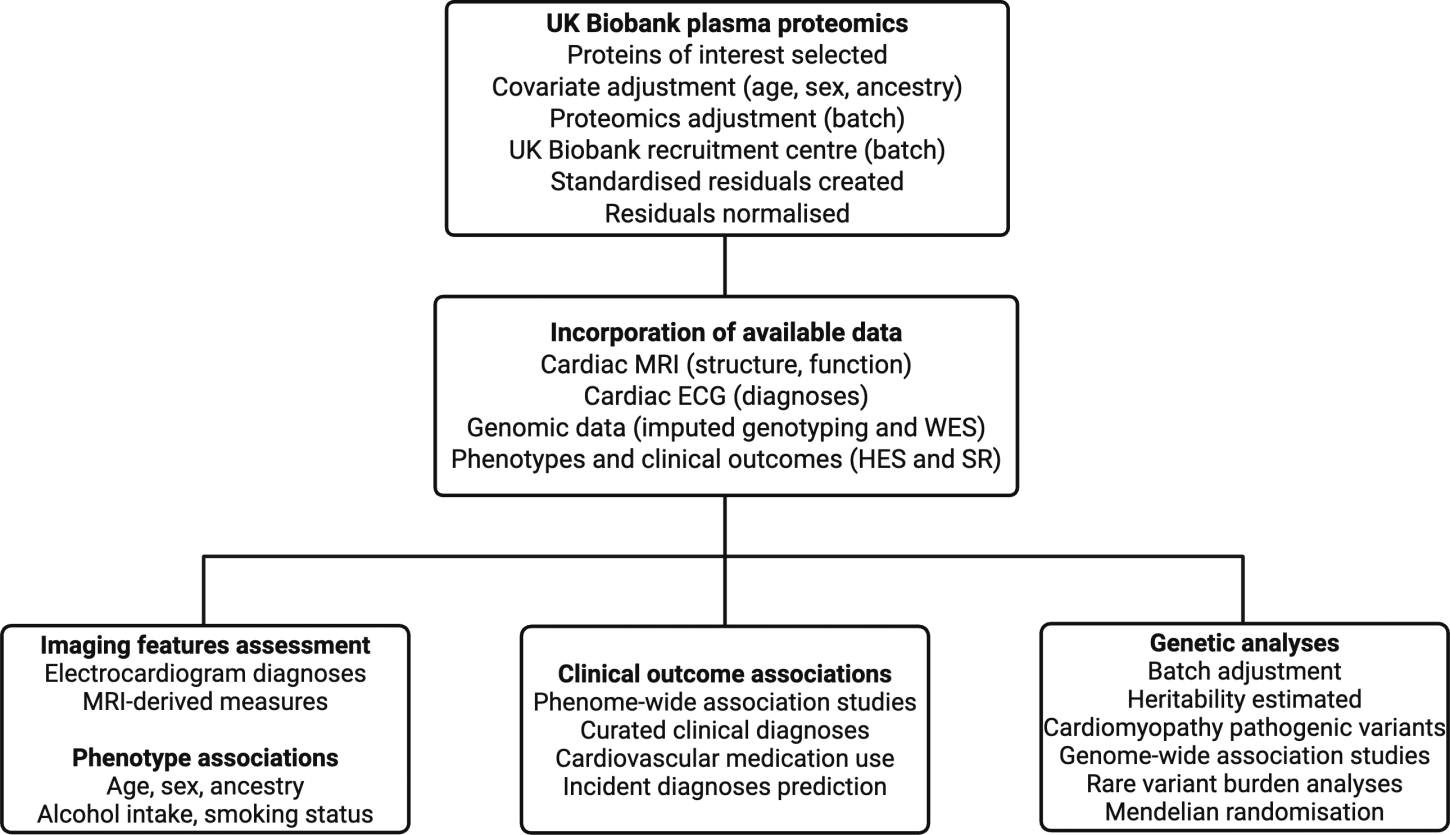
**Study flow chart and protein summary.** A summary of the main analysis steps and data available for the analysis of 9 plasma proteins and the genetic and outcome associations. HES indicates hospital episode statistics; MRI, magnetic resonance imaging; WES, whole exome sequencing; and SR, self-reported.

Genotyping array data and exome sequencing data were available for over 450 000 participants. Substudies analyzed baseline plasma proteomics in over 45 000 participants^12^ and recalled participants for ECGs^13^ and cardiac magnetic resonance imaging.^14,15^ Additional phenotypic and outcome data included hospital episode statistics, self-reported questionnaire data, alcohol intake,^16^ and smoking status. Protein levels were assessed for association with genetic, phenotypic, and clinical outcome data (Figure 1). Two-sample Mendelian randomization was used to determine causality, and risk prediction of Cox proportional hazards regression models was created to understand whether the biomarkers predicted incident diagnoses.

### Proteomics

Plasma from the initial UKB assessment visit (2006–2010) was collected. Details of participant randomization, sample handling, Olink proteomics assay through the antibody-based Olink Explore 3072 PEA, data processing, and quality control are as detailed previously.^12^ Briefly, proteomics was undertaken on samples of a randomly selected subset of 46 595 UKB participants at the baseline visit. The raw protein levels are provided in normalized protein eXpression; Olink’s arbitrary unit in log_2_ scale.

### Genetic Analyses

Genotyping array data and exome sequencing data were available for over 450 000 participants. Genotype calling and exome sequencing were performed and imputed as described previously.^17,18^ Individuals with proteomics data were extracted, and GWAS was undertaken using GCTA software (version 64; Table S1; Figure S1).^19^ A sparse genetic relationship matrix was created, and FastGWA was undertaken with a mixed linear model, adjusting for the genotyping array batch. Genes of independent loci were prioritized through LocusZoom and expression quantitative trait loci from the Genotype-Tissue Expression project (v8). Phenotype associations through the GWAS catalog and PheWEB were assessed. Heritability was estimated by creating a genetic relationship matrix in GCTA and using a restricted maximum likelihood analysis to estimate the variance explained by the SNPs that were used to estimate the genetic relationship matrix. Mendelian randomization was undertaken using GWAS summary statistics from published literature^5,6,20,21^ using the R package TwoSampleMR. Exposure variants were included if the GWAS was significant (*P*<5×10^−8^). Tests of pleiotropy, Steiger directionality, and heterogeneity were assessed (Supplemental Methods).

Rare variant association studies (RVAS) were undertaken using Regenie software on the DNA Nexus Research Analysis Platform.^22^ The genotyping data were used for step 1 of Regenie, and exome sequencing data for step 2. Step 2 was run over different allele frequencies (singletons, 0.01, 0.001) for 6 overlapping, protein-altering variant, custom masks (loss-of-function only; missense only [flagged by >1 of 5 deleterious software]; missense only [all]; missense only [flagged by all of 5 deleterious software]; protein-altering variants [loss-of-function and missense flagged by >1 of 5 deleterious software]; protein-altering variants [all loss-of-function and missense]), where the minimum minor allele count was at least 3. Bonferroni significance for 18 117 included genes was *P*<2.76×10^−6^.

Cardiomyopathy-associated rare variants were identified as previously published^5,23,24^ for hypertrophic (HCM) and dilated cardiomyopathy (DCM). Individuals were classified as genotype negative if they had no rare protein-altering genetic variation (minor allele frequency <0.001 in the UKB and the Genome Aggregation Database) in any genes that may cause or mimic HCM or DCM. These genes represented an inclusive list of genes with definitive or strong evidence of an association with cardiomyopathy, moderate evidence, and genes associated with syndromic phenotypes.^1,4,11^ This genotype-negative group was compared with individuals with disease-associated rare variants in genes with strong or definitive evidence for HCM (*MYBPC3, MYH7, MYL2, MYL3, TNNI3*, *TNNT2*, *TPM1*, and *ACTC1*) and DCM (*BAG3*, *DES*, *DSP*, *FLNC*, *LMNA*, *MYH7*, *PLN*, *RBM20*, *SCN5A*, *TNNC1*, *TNNT2*, and *TTN*). Analysis was restricted to robustly disease-associated variant classes for each gene^4,11^ and to variants sufficiently rare to cause penetrant disease (filtering allele frequency <0.00004 for HCM and 0.000084 for DCM^25^). Variants were classified as pathogenic/likely pathogenic (sarcomeric) if reported as pathogenic/likely pathogenic for cardiomyopathy in ClinVar and confirmed by manual review.

### Cardiac MRI and ECG Analyses

A substudy recalled participants for imaging, including cardiac magnetic resonance,^15^ and ECG. For cardiac magnetic resonance, volumetric traits were measured using quality-controlled deep learning algorithms.^14^ Deep neural networks were used for short-axis cine segmentation via a fully convolutional network to label pixels containing myocardium. The performance of image annotation using this algorithm is equivalent to a consensus of expert human readers and achieves subpixel accuracy for cardiac segmentation.^14^ Five thousand three hundred twenty-four participants with proteomics had imaging data available.

The ECGs were performed according to a defined protocol and analyzed using proprietary software (GE CardioSoft, Boston, MA). Data from the first imaging visit (instance 2, n=42 386) was labeled using a previously trained convolutional neural network designed to identify 6 diagnoses from the ECG^13^ sinus bradycardia, sinus tachycardia, left bundle branch block, right bundle branch block, first degree AV block, and atrial fibrillation. The binary outputs (presence or absence of each diagnosis) were used for subsequent analyses. 4831 participants with proteomics had ECG data available.

For analyses comparing plasma proteomics sampled at recruitment to records from the subsequent imaging appointment, sensitivity analyses also included an adjustment for the difference in time.

### Statistical Analyses

The analyses were undertaken using R (v4.1.2) and the UKB research analysis platform. The proteomic measures were adjusted using multiple linear regression for age at recruitment, age,^2^ UKB recruitment centers, genetically determined sex, age×sex interaction, proteomics batch, and genetically determined European ancestry, and the resulting standardized residuals (mean=0, SD=1) were normalized by an inverse rank normalization. Cardiovascular-associated medication intake was self-reported and curated for participants who reported taking medications of interest (Table S2). Phenome-wide association studies were undertaken using the phenome-wide association studies R package with clinical outcomes and coded phenotypes converted to 1840 categorical PheCodes. *P* values were deemed significant with Bonferroni adjustment for the number of PheCodes measured.

Cox proportional hazards regression models were assessed with the full cohort of participants and created using the first reported UKB data (summarizing the first date of a report from all UKB data [Hospital episode statistics, primary care, self-reported, etc]) for heart failure, cardiomyopathy, atrial fibrillation, hypertension, diabetes, and myocardial infarction, as identified through phenome-wide association studies, using the survival and survminer R packages by age to death, diagnosis, or last date of follow-up report. Participants diagnosed before recruitment were excluded. Participants who died without a diagnosis were also excluded as the sensitivity analyses.

## Results

### Circulating Biomarkers of Age, Sex, Alcohol Intake, Smoking Status, and Ancestry

Forty-six thousand eleven participants had measures of the proteins assessed (participant characteristics: Table S1). The correlation between the levels of NT-proBNP and BNP was *R*=0.67 (Figure S2). Levels of ACTA2 had the strongest relationships with the other circulating protein levels (eg, ACTN4 (*R*=0.30), NT-proBNP (*R*=0.30), BNP (*R*=0.21), BAG3 (*R*=0.21); Figure S2).

A positive relationship was identified with age at recruitment for measures of ACTA2 (*R*=0.42), NT-proBNP (*R*=0.34), BNP (*R*=0.23), and ACE2 (*R*=0.16; Figure S3). ACE2, CDKN1A, and TNNI3 were increased in male compared with female participants (protein level mean difference β=0.47, *P*=1.0×10^−16^; β=0.20, *P*=6.61×10^−68^; β=0.21, *P*=3.97×10^−127^; respectively; Figure S4). NOTCH1, NT-proBNP, and BNP were increased in female participants (β=0.06, *P*=8.61×10^−258^; β=0.42, *P*=1.62×10^−278^; β=0.25, *P*=3.50×10^−69^; respectively; Figure S4).

Relationships were observed between alcohol intake and smoking status (smoker at recruitment compared with never smoked), and measures of ACE2 (*R*=0.14; β=0.22, *P*=1.70×10^−44^; respectively) and NOTCH1 (*R*=−0.15; β=−0.16, *P*=6.11×10^−23^; respectively). BAG3, CDKN1A, and TNNI3, also had relationships with smoking status (β=−0.08, *P*=7.94×10^−8^; β=0.10, *P*=2.38×10^−10^; β=−0.05, *P*=0.0008; respectively).

**Figure 2.**
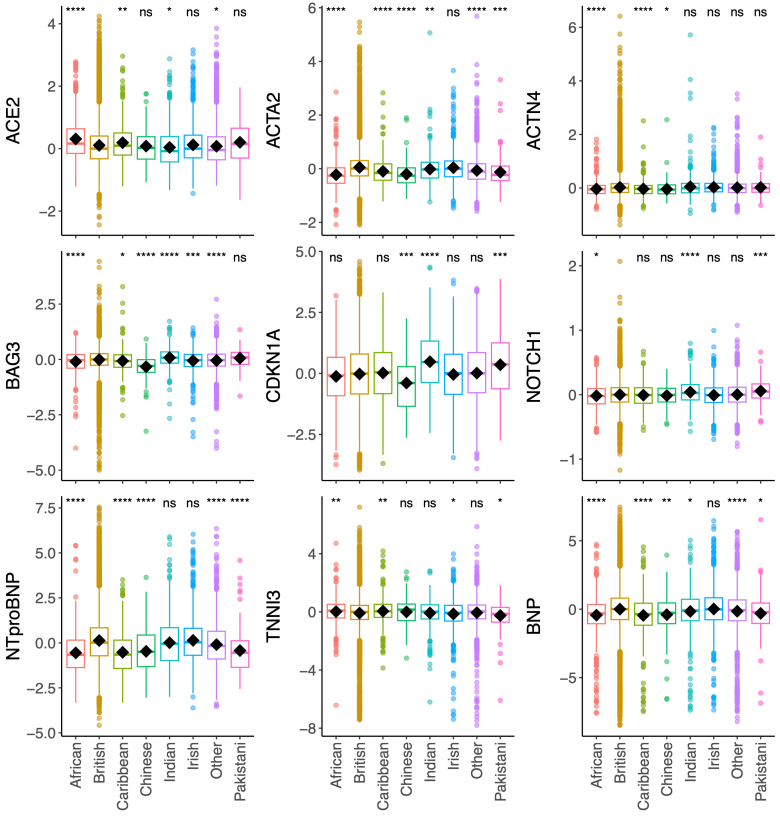
**The relationships between the proteins and ancestry.** The plots depict the significant differences in protein levels across self-reported ancestries. Participants of self-reported African or Caribbean ancestry had increased average ACE2 (angiotensin-converting enzyme 2) and decreased ACTA2 (actin alpha 2), ACTN4 (actinin alpha 4), BAG3 (BAG cochaperone 3), NT-proBNP (N-terminal pro-B-type natriuretic peptide), and BNP (B-type natriuretic peptide), compared with British ancestry. Participants with Chinese ancestry had decreased average ACTA2, BAG3, CDKN1A (cyclin-dependent kinase inhibitor 1A), and NT-proBNP, and participants with Indian ancestry had increased average BAG3, CDKN1A, and NOTCH1 (neurogenic locus notch homolog protein 1), compared with British ancestry. The significance of differences in means as derived by the Student *t* test is denoted as stars compared with British ancestry. The *y* axis units are Olink arbitrary units in log_2_ scale. The sample sizes were as follows (African, n=584; British, n=40228; Caribbean, n=434; Chinese, n=131; Indian, n=495; Irish, n=1200; other, n=2736; and Pakistani, n=143). TNNI3 indicates troponin I.

Proteomic variability was observed with ancestry. Participants of self-reported African or Caribbean ancestry (n=584 and n=434, respectively) had increased average ACE2 and decreased ACTA2, ACTN4, BAG3, NT-proBNP, and BNP compared with British ancestry, which dominates the UKB cohort (n=40 228, 87% British; Figure 2). Participants of Chinese ancestry (n=131) had decreased average ACTA2, BAG3, CDKN1A, and NT-proBNP, and participants of Indian ancestry (n=495) had increased average BAG3, CDKN1A, and NOTCH1, compared with British ancestry (Figure 2).

### Medication Use

Medication use was reported at recruitment when the blood was sampled for proteomics (n=46 011 participants) and 8.4 years later (range 3.8–12.7 years) at the imaging appointment (n=5324). Of the individuals with reported cardiovascular medications at recruitment, 39% to 75% also reported the medication at the imaging appointment (Supplemental Results).

Most of the protein levels (measured from plasma samples collected at recruitment) had significant associations with medication use reported at recruitment (Table S2). To understand whether the protein levels may herald a disease that will require future treatment, protein measures at recruitment were assessed for an association with medication reported at the imaging visit. Participants with increased ACE2, NT-proBNP, and BNP levels at recruitment were more likely to report β-blocker use at the imaging visit 8 years later. ACE2 was also associated with ACE inhibitor (β=0.27; *P*=7.90×10^−11^) and angiotensin receptor blocker use (β=0.22; *P*=8.44×10^−4^), and participants with increased NT-proBNP or BNP at recruitment were more likely to report anticoagulant use (β=1.02, *P*=5.44×10^−6^; β=0.91, *P*=6.07×10^−6^; respectively) at the imaging visit (Table S2). The results were similar when the proteins were adjusted for the time between recruitment and the imaging visit (Table S2; Figure S5).

**Figure 3.**
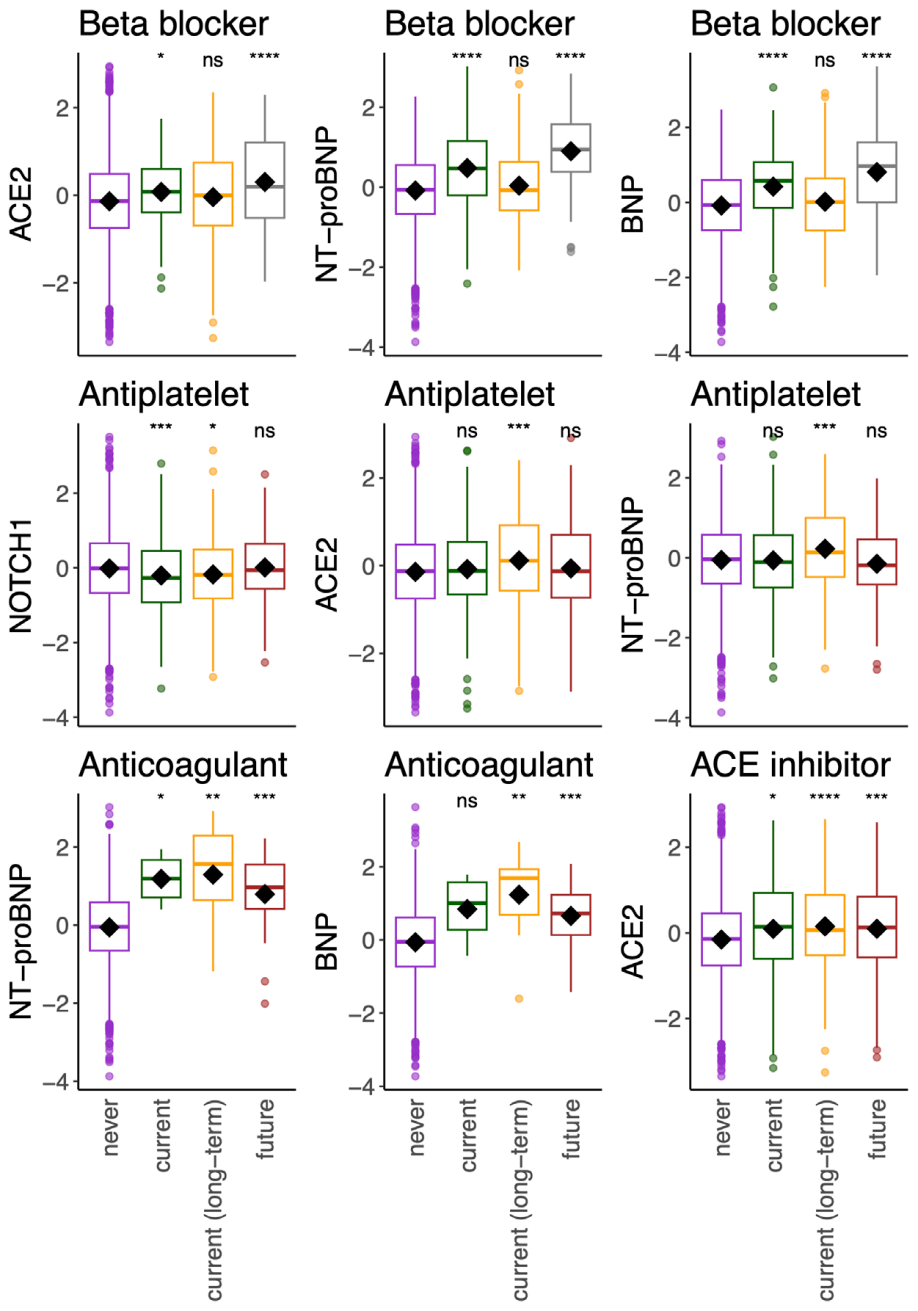
**The relationships between the proteins and medication intake.** The plots depict the proteins measured at recruitment that were significantly increased with medication intake reported only at recruitment (current), or the imaging visit on average 8 years later (future), or reported during both visits (current [long-term]). The significance of differences in means as derived by the Student *t* test is denoted by stars compared with no report of the medication (never). The *y* axis units are standardized residuals after adjustment for covariates. The data only includes those with proteomics who attended the imaging visit (n=5324). ACE2 indicates angiotensin-converting enzyme 2; BNP, B-type natriuretic peptide; NOTCH1, neurogenic locus notch homolog protein 1; and NT-proBNP, N-terminal pro-B-type natriuretic peptide.

To further understand whether the increase in plasma proteins at recruitment was due to medication use at recruitment or predictive of future medication use, we assessed the change in medication use: whether the protein levels at recruitment were significantly altered with medication (1) reported only at recruitment, (2) uptake by the imaging visit, or (3) longer-term use reported during both visits, compared with participants without the reported medication (Figure 3). ACE2 levels predicted the future uptake of β-blockers and ACE inhibitors, suggesting that ACE2 plasma levels herald prescription and associated diseases. NT-proBNP and BNP levels predicted long-term use and future uptake of anticoagulants (Figure 3). The increase in NT-proBNP and BNP levels observed for β-blockers may be in part due to the use of the respective medications and overt disease at recruitment, as they predicted both current and future use.

### Association With Clinical Features and Diagnoses

**Figure 4.**
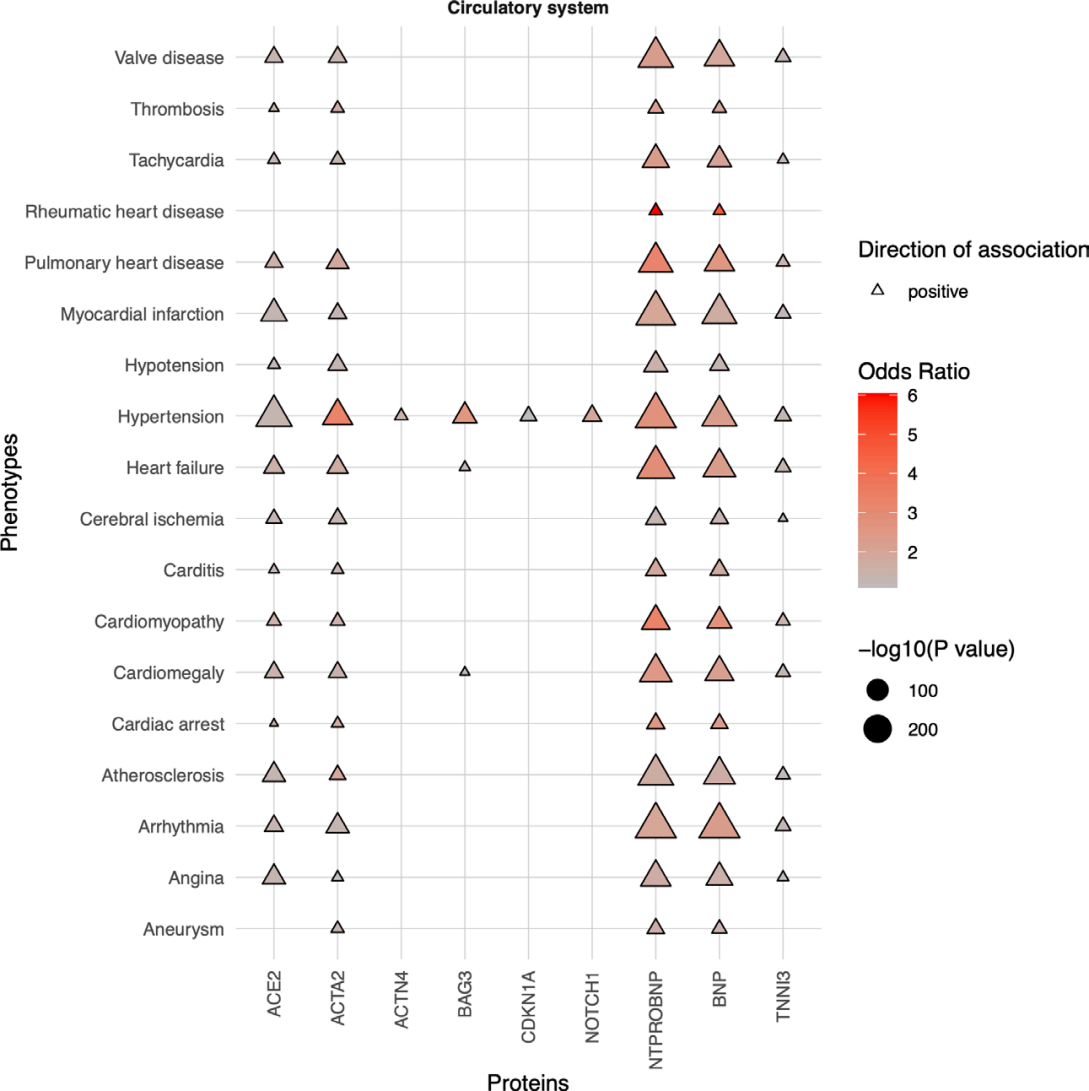
**Phenome-wide association study results of the plasma protein levels with selected circulatory disorders.** Phenotypes as phecodes are described on the *y* axis and the protein traits on the *x* axis. Each point denotes a significant phenome-wide association study (PheWAS) association with a Bonferroni correction for the number of analyzed phecodes. The shape and color denote the direction of effect and the odds ratio. Only the most significant associations with selected, nonredundant phenotypes of the circulatory disorder category are presented for clarity. See Table S3 for the full PheWAS results. As no negative direction of association was identified, points do not have the shape of an inverted triangle. ACE2 indicates angiotensin-converting enzyme 2; ACTA2, actin alpha 2; BAG3, BAG cochaperone 3; BNP, B-type natriuretic peptide; CDKN1A, cyclin-dependent kinase inhibitor 1A; NOTCH1, neurogenic locus notch homolog protein 1; NT-proBNP, N-terminal pro-B-type natriuretic peptide; and TNNI3, troponin I.

Through phenome-wide association studies, the most significant associations with each protein were with cardiac dysrhythmias (NT-proBNP), atrial fibrillation and flutter (BNP), chronic renal failure (ACTA2, BAG3, NOTCH1, ACTN4), type-2 diabetes (ACE2, CDKN1A), and congestive heart failure (TNNI3; Figure 4; Table S3; Figures S6 through S20). Renal diseases associated with most of the protein measures analyzed (Table S3). ACE2, ACTA2, NT-proBNP, and BNP levels, associated with a range of respiratory conditions (Table S3). ACE2 levels are also associated with type-2 diabetes, epilepsy, tobacco use disorder, alcohol-related disorders, and liver diseases (Figures S6 through S20; Table S3).

A curated analysis was used to assess associations between the 9 circulating proteins and specific diagnoses of cardiomyopathies, muscular dystrophy, heart failure, scoliosis, respiratory failure, coronary disease, cardiac arrhythmia (including atrial fibrillation and flutter), stroke, hypertension, valve disease, hypercholesterolemia, and diabetes (Figure S21; Table S4). ACE2, ACTA2, TNNI3, NT-proBNP, and BNP levels had the most associations with curated traits. Hypertension and heart failure correlated with all proteins, except NOTCH1 and CDKN1A, respectively.

### Cardiac ECG Diagnoses and MRI Parameters

Participants with increased NT-proBNP at recruitment were diagnosed more frequently with sinus bradycardia (protein level mean difference, β=0.25, *P*=2.82×10^−7^) and atrial fibrillation (β=0.92, *P*=7.24×10^−9^) on future ECGs. The associations remained significant when individuals with reported β-blocker use were removed from the analyses (Supplemental Results). The association with atrial fibrillation was also observed for BNP (β=0.81, *P*=2.27×10^−6^). Adjustment for the time between recruitment and imaging had little effect (Supplemental Results).

Analyses of cardiac magnetic resonance-derived traits identified relationships between NT-proBNP and BNP and increased left atrial volume (*R*=0.14–0.17) and decreased atrial ejection fraction (*R*=−0.14 to −0.19). NT-proBNP also correlated with decreased ventricular wall thickness (*R*=−0.21) and volumes (*R*=−0.11 to −0.18) and increased ventricular ejection fraction (*R*=0.12). The relationships between NT-proBNP and BNP with atrial volumes (*R*=0.11–0.21) and with atrial ejection fractions (*R*=−0.11 to −0.17) were not affected by an adjustment for the time between the recruitment and imaging visit (Table S5).

### Carriers of HCM and DCM Pathogenic Variants

Participants carrying a pathogenic/likely pathogenic variant in a HCM-associated gene had increased levels of ACE2 and NT-proBNP (but not BNP) at recruitment (β=0.47, *P*=0.0004; β=0.62, *P*=0.0007; respectively). Participants carrying a pathogenic/likely pathogenic variant in a DCM-associated gene also had increased NT-proBNP at recruitment (β=0.32, *P*=0.0002). These associations may be due to overt cardiomyopathy, as the signals became nonsignificant with the removal of individuals with diagnosed cardiomyopathy.

### Genetic Association Studies

SNP-based heritability of the protein levels, the amount of variation in the protein levels estimated to be due to genetic factors, identified ACE2 levels with the highest estimated heritability (34.5%), followed by NT-proBNP (33.5%), BAG3 (25.7%), NOTCH1 (22.3%), BNP (18.9%), ACTA2 (17.0%), and CDKN1A (14.2%). ACTN4 and TNNI3 levels had very low and nonsignificant heritability estimates; either more influenced by nongenetic factors or measurement error.

**Figure 5.**
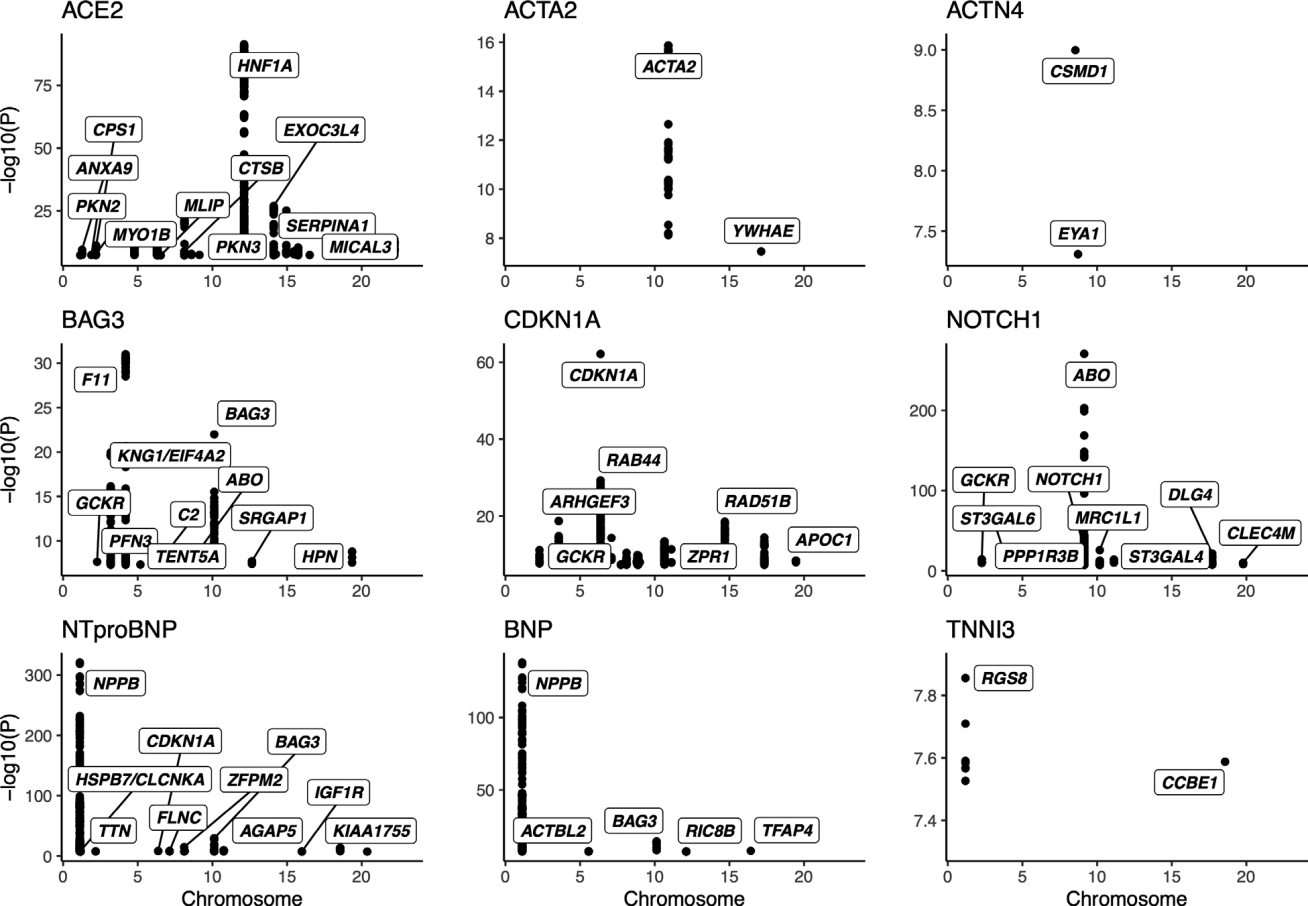
**Significant genome-wide association study results.** The Manhattan plots present the genome-wide association study (GWAS) significant SNPs for the 9 protein levels. The prioritized gene is noted for the significant loci identified. A subset of gene labels for ACE2 (angiotensin-converting enzyme 2), CDKN1A (cyclin-dependent kinase inhibitor 1A), and NT-proBNP (N-terminal pro-B-type natriuretic peptide) has been selected to allow for presentation. The *y* axis is cut at a minimum of 5×10^−8^. Please see Table S6 for the full GWAS results. ACTA2 indicates actin alpha 2; BAG3, BAG cochaperone 3; BNP, B-type natriuretic peptide; CDKN1A, cyclin-dependent kinase inhibitor 1A; NOTCH1, neurogenic locus notch homolog protein 1; NT-proBNP, N-terminal pro-B-type natriuretic peptide; and TNNI3, troponin I

To identify genetic modifiers of the circulating protein levels, we undertook a GWAS (Figure 5; Tables S1 and S6; Figure S1) and an RVAS (Tables S1 and S7) for each protein measure. The GWAS and RVAS of the levels of ACTA2, BAG3, CDKN1A, NOTCH1, NT-proBNP, and BNP identified the expected gene-protein pair (cis-expression quantitative trait loci were identified at the genetic loci of *ACTA2*, *BAG3*, *CDKN1A*, *NOTCH1*, and *NPPB*).

Recurrent modifiers included *GCKR* for BAG3, CDKN1A, and NOTCH1 levels, *APOE* for CDKN1A and NOTCH1 levels, and *SERPINA1* for ACE2 and NOTCH1 levels (Table S6). Glucokinase regulator (*GCKR*) is a regulatory protein that inactivates glucokinase in liver and pancreatic islet cells and has been previously associated with hyperlipidemia and diabetes. Apolipoprotein E (*APOE*) is an apolipoprotein involved in lipoprotein metabolism. Serpin family A member 1 (*SERPINA1*) is a serine protease inhibitor associated with alpha 1-antitrypsin deficiency. Targets of SERPINA1 include elastase, plasmin, thrombin, trypsin, chymotrypsin, and plasminogen activator. The gene is linked to chronic obstructive pulmonary disease, emphysema, and chronic liver disease. The lead variant is associated in previous GWAS with varied traits, including lipid metabolism (rs28929474; eg, Karjalainen et al^26^).

RVAS found that ACTA2 levels were associated with missense variants in *LMOD1*. Leiomodin 1 (*LMOD1*) has been implicated in smooth muscle dysfunction and thoracic aortic aneurysm and dissection previously and is predicted to interact with ACTA2.^27^

### ACE2 and Its Potential Role in Hypertension and Diabetes

**Figure 6.**
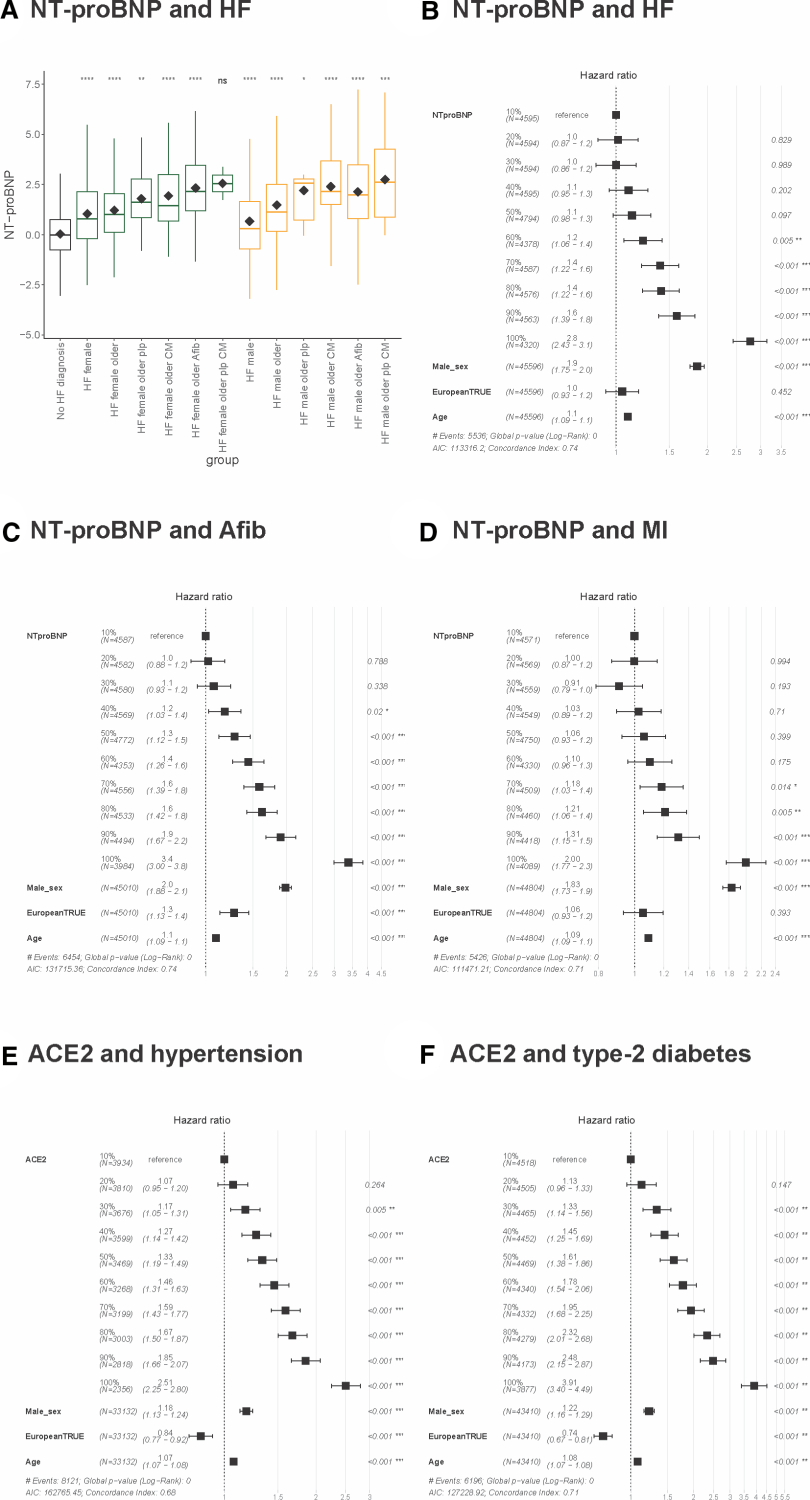
**Increasing levels of NT-proBNP (N-terminal pro-B-type natriuretic peptide) and ACE2 (angiotensin-converting enzyme 2) are observed with cardiovascular disease diagnoses.** Increasing levels of NT-proBNP (N-terminal pro-B-type natriuretic peptide) are observed in participants diagnosed with incident heart failure (HF), atrial fibrillation (Afib), and myocardial infarction (MI). Increasing levels of ACE2 (angiotensin-converting enzyme 2) are observed in participants diagnosed with incident hypertension and type-2 diabetes. **A**, The figure shows the sequential increase in mean NT-proBNP with overt diseases and other modifiers influencing the protein’s levels. This includes a HF diagnosis alongside sex, age at recruitment, a diagnosis of cardiomyopathy (CM) or Afib, and carriers of pathogenic CM-associated variants. The Student *t* test was used to compare with the no HF diagnosis reference group. The groups contained the following sample sizes, respectively: no HF diagnosis, 44 050; female groups, 103, 286, 12, 33, 234, 2; male groups, 202, 496, 7, 42, 470, 14. NT-proBNP units are Olink’s arbitrary unit on log_2_ scale. The forest plots (**B–F**) of Cox proportional hazards regression models assessed death or diagnosis from recruitment, with those diagnosed before recruitment excluded. Sex (increasing risk is male), European ancestry (increasing risk is European), and age at recruitment (incremental risk per year lived) were added to this multivariable analysis for comparison. Forest plots are presented for deciles of NT-proBNP levels with incident (**B**) HF, (**C**) Afib, (**D**) MI, from recruitment, and ACE2 levels by decile with incident (**E**) hypertension and (**F**) type-2 diabetes, from recruitment. Plp indicates P/LP variant carrier.

**Figure 7.**
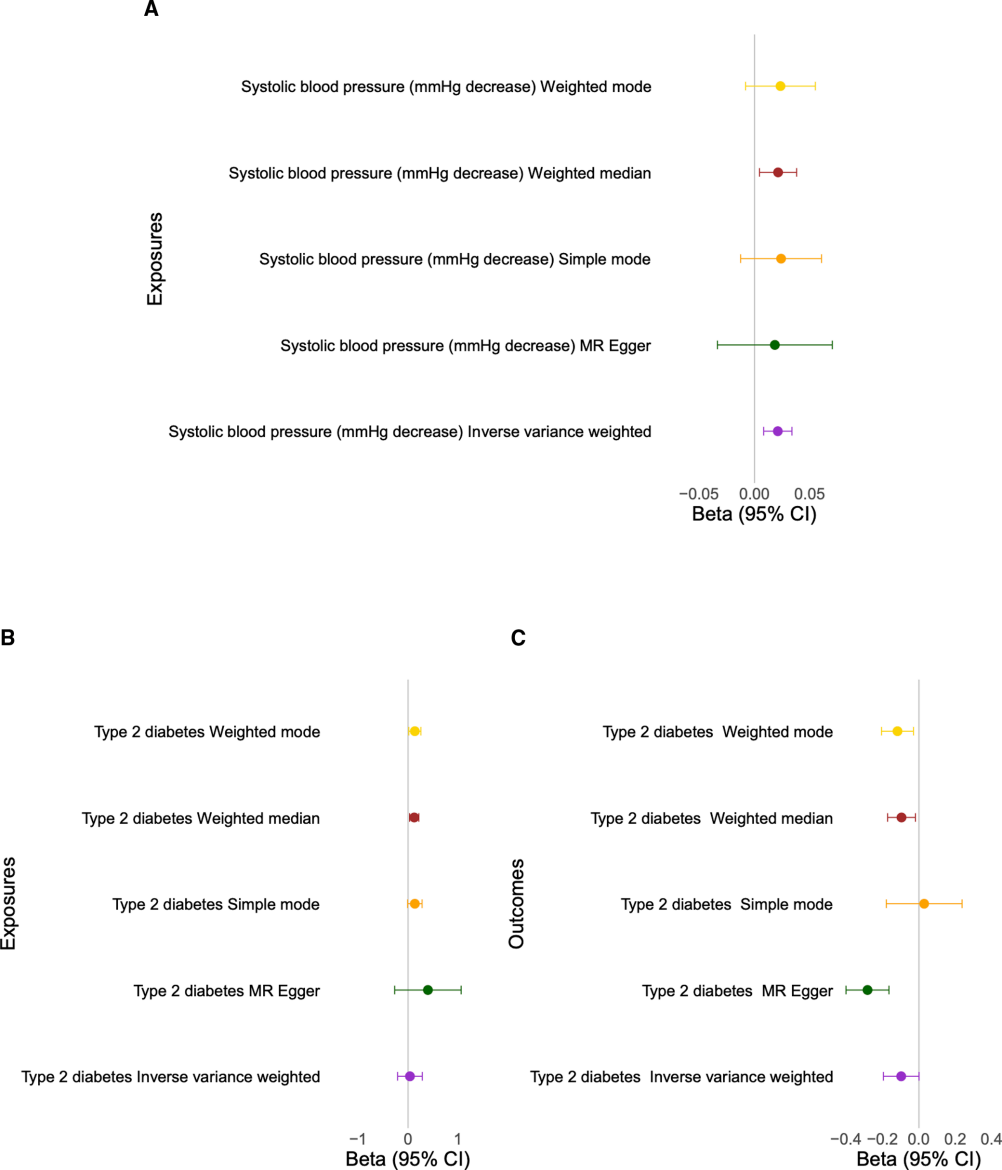
**Evidence of a causal relationship between systolic blood pressure, type-2 diabetes, and ACE2 (angiotensin-converting enzyme 2).** Increased circulating ACE2 can decrease blood pressure through the creation of vasodilators and is causally associated with type-2 diabetes. A genetic predisposition for decreased systolic blood pressure is associated with increased ACE2. **A**, Mendelian randomization (MR) genetic determination model of systolic blood pressure (mm Hg decrease), genetic instruments as exposures for the ACE2 outcome. Two-sample MR was undertaken with ACE2 (using the GWAS [genome-wide association study] results) and decreased systolic blood pressure (from GWAS summary statistics of published data). **B**, MR genetic determination model of type-2 diabetes genetic instruments as exposures for the ACE2 outcome. Two-sample MR was undertaken with ACE2 (using the GWAS results) and type-2 diabetes (from GWAS summary statistics of published data). **C**, MR genetic determination model of the ACE2 genetic instrument as an exposure for type-2 diabetes. See Table S8 and Figures S3 and S4 for further details.

ACE2 is found on the X-chromosome, and we identified increased ACE2 levels in males compared with females (β=0.47, *P*=1.0×10^−16^). Increasing ACE2 levels at recruitment were positively correlated with systolic blood pressure (*R*=0.21; Figure S21) and were predictive of an incident hypertension diagnosis (Figure 6; Figure S22). These associations were more significant in females (*R*=0.20 in females and *R*=0.15 in males; Figure S22, respectively). Two-sample Mendelian randomization with a genetic instrument for systolic blood pressure^20^ showed evidence for a protective, compensatory relationship: instruments associated with decreased systolic blood pressure increased circulating ACE2 (Figure 7; Figures S23 and S24; Table S8). This suggests a protective role for increased ACE2 levels in blood pressure control, validating the findings of published loss- and gain-of-function mouse model experiments.^28^

We showed that ACE2 was significantly increased on average in participants of African or Caribbean ancestry, and some guidelines suggest ACE inhibitors (targeting the ACE1 protein) are less effective in Black individuals (see Discussion). A high level of ACE2 at recruitment in participants of African or Caribbean ancestry was not significantly predictive of a hypertension diagnosis; however, a limitation of this experiment is the small sample size (n=682).

ACE2 levels were associated with the loci of diabetes-associated genes *HNF1A* and *HNF4A* at GWAS and RVAS (Tables S6 and S7). Increasing ACE2 levels at recruitment were predictive of an incident type-2 diabetes diagnosis, particularly in females (Figure 6; Figures S16 and S22). Two-sample Mendelian randomization with the results of a GWAS of type-2 diabetes^29^ showed evidence for an inverse relationship: ACE2 genetic instrument decreased the risk of type-2 diabetes, whereas type-2 diabetes increases ACE2 plasma levels (Figure 7; Figure S25).

### NT-proBNP and BNP and Their Role in Heart Failure

The GWAS of NT-proBNP levels identified cardiomyopathy- or heart failure-associated genetic loci (eg, *HSPB7/CLCNKA*, *CDKN1A*, *TTN*, *FLNC*, and *BAG3*). The GWAS of BNP levels also identified an association with variants in the loci of *BAG3*. Through RVAS, NT-proBNP, and BNP were associated with variants in *NPPB* and the region surrounding the natriuretic peptide genomic locus (*NPPA/NPPB*). This suggests that the natriuretic peptide locus is the major effect locus of NT-proBNP and BNP levels, as expected.

NT-proBNP and BNP levels were increased in individuals with overt cardiomyopathy (Figure S21) and were predictive of incident diagnoses (heart failure, cardiomyopathy, and atrial fibrillation; Figure 6; Figures S22, S26, and S27). To further understand the relationship with cardiomyopathies, we undertook 2-sample Mendelian randomization with the results of GWAS of HCM^30^ and DCM.^5^ This analysis suggested that left ventricular hypertrophy increases NT-proBNP and BNP circulating levels (Figures S28 and S29; Table S8).^10,21,31–33^ The opposite effect was observed for the DCM Mendelian randomization; common genetic variants associated with DCM risk^5^ predisposed to decreased NT-proBNP levels (Figure S28; Table S8). Opposing genetic relationships between HCM and DCM common variants have been described elsewhere^7,30^ and indicate that genetic loci underlying the variability of left ventricular function in the general population may be differentially involved in susceptibility to HCM and DCM.

NT-proBNP had a positive relationship with a heart failure diagnosis alongside older age, a diagnosis of cardiomyopathy or atrial fibrillation, and carriers of pathogenic cardiomyopathy-associated variants (Figure 6; Figures S22, S27, S30, and S31; Supplemental Results). We showed that NT-proBNP and BNP were significantly decreased on average in participants of African or Caribbean ancestry. Increasing NT-proBNP levels at recruitment in participants of African or Caribbean ancestry remained significantly predictive of heart failure (BNP was not significant).

## Discussion

We identified relationships between circulating proteins with cardiovascular disease and those that mediate causal relationships between cardiovascular risk factors and disease development. Genetic studies identified variants in *GCKR*, *APOE*, and *SERPINA1*, as modifiers for >1 cardiovascular-associated protein. Six of the 9 proteins assessed have been previously identified^34^ as the strongest predictors in large-scale proteomic risk scores,^31^ particularly NT-proBNP (Supplemental Discussion).

### ACE2 and Hypertension, Diabetes, and COVID-19

It has been previously shown that circulating ACE2 is a prominent predictor of cardiovascular outcomes,^32^ and it has potential as a biomarker and measure of cardiovascular disease risk. This may be due to a soluble form of ACE2 released from cell membranes into the circulation, resulting in a loss of ACE2 and renin angiotensin aldosterone pathway vasodilation in tissues, but an increased ACE2 blood measurement.^28^ Through 2-sample Mendelian randomization, we showed that genetic instruments for decreased systolic blood pressure correlated with increased circulating ACE2 levels. The relationships described here of ACE2 circulating levels with cardiovascular risk mediators and genetic factors suggest that it may be an important target for therapeutic intervention for cardiovascular disease, particularly in females.

Teasing apart the role of the increase in vasodilator ACE2 effects in balance with vasoconstrictor ACE1 effects would aid our understanding of the success of specific ACE inhibitors, whether the upregulation or stimulation of ACE2-induced vasodilation would be beneficial in clinical practice, and whether the selective treatment of specific ACE inhibitors known to influence ACE2^33^ would be beneficial for patients with altered circulating ACE2 levels. Further understanding is required of the balance between ACE and ACE2 activity in determining cardiovascular outcomes. It is not clear to what extent their counterbalanced activities are mutually exclusive in homeostasis, but it has been suggested that an activity imbalance progresses COVID-19-related disease and the renin angiotensin aldosterone pathway.^28^ There have been extensive studies of ACE2 in COVID-19 (where the COVID-19 virus was found to engage ACE2 for cellular entry), including the identification of the upregulation of ACE2 with smoking^34,35^ and alcohol.^36–38^

ACE inhibitors are thought to have less blood pressure–lowering effects and increased risk of angioedema in Black individuals with hypertension, and international guidelines preferentially recommend diuretics and calcium channel blockers over ACE inhibitor treatment in these individuals. Concerns have been expressed with the generalisability of the guidelines and the lack of mechanistic understanding.^39^ Further exploration is required into the increased average circulating ACE2 protein levels observed here in a small number of individuals of self-reported African or Caribbean ancestry, particularly concerning confounding factors, such as hypertension prevalence. Targeted proteomics in cohorts of African ancestry would be of interest here.

ACE2 stimulation as a therapeutic strategy may be particularly relevant for individuals exhibiting limited treatment efficacy from certain ACE inhibitors and angiotensin receptor blockers, and systemic delivery of recombinant human ACE2 may be an alternative approach. Current clinical trials of recombinant human ACE2 focus on neutralizing COVID-19 (clinicaltrials.gov) and activating the systemic protective axis of the renin angiotensin aldosterone pathway.^28^ However, it is unknown whether the suggested detrimental release of soluble ACE2 from cells can be attenuated through systemic ACE2 delivery to improve cardiovascular outcomes.

Angiotensin receptor blockers prevent the action of angiotensin II for high blood pressure regulation, preventing heart failure, and treating kidney failure in people with diabetes. *ACE2* increased expression in the endocrine pancreas in diabetes is hypothesized to act in a compensatory manner,^40^ and here, we provide evidence of genetic associations that strengthen a potential role for *ACE2* expression and protection against diabetes. Conversely, as a biomarker, ACE2 may have potential as a measure of increased cardiovascular disease risk.

### NT-proBNP: A Predictive Biomarker of Hypertrophic Cardiomyopathy

NT-proBNP is a prohormone with an N-terminal that is cleaved to release brain or BNP (Supplemental Discussion). We identified an opposing relationship between HCM and DCM and the BNPs (both NT-proBNP and BNP); although the levels of BNPs are increased with the progression of both cardiomyopathies, causality via Mendelian randomization suggested that the BNPs are part of HCM pathology and progression, whereas the observed increase in DCM is an adaptive response to contractile dysfunction and cardiomyocyte stretch. The roles of the BNPs in natriuresis and promoting hypertrophy may protect against DCM-associated systolic dysfunction. Endogenous natriuretic peptides protected the heart in a mouse model of DCM and sudden death. *NPPB* knockout rats at 3 months showed hypertrophy without alteration to the ventricles or function and this transitioned at 6 months into DCM. Diabetic cardiomyopathy mouse models treated with exogenous BNP prevented the development of DCM, whereas knockdown of endogenous BNP accelerated DCM.^41–43^

Angiotensin receptor blockers have been trialed as a treatment for early-stage HCM^44^ based on data that suggest they abrogated the development of hypertrophy and fibrosis. Initial results of the VANISH trial were encouraging. Other studies are investigating the combination of sacubitril-valsartan^45^ (eg, NCT04164732). Sacubitril is a neprilysin inhibitor that inhibits the degradation of BNPs. Our results question whether this combination may have negative consequences in patients with hypertrophy, given the possible effects of increased BNP in this context. Our results are in keeping with existing data that support a positive effect in patients with heart failure with reduced ejection fraction,^46^ including those with DCM. Further studies are required to address this.

We showed better predictive capacity for measures of circulating NT-proBNP than BNP with cardiovascular disease. Although the measures are highly correlated (*R*=0.67), there are important differences in measurable levels: NT-proBNP is measured at a higher concentration, has a higher prognostic value,^47^ has been shown to have sustained elevation for 12 weeks,^48^ the predictive capacity of the ratio of NT-proBNP:BNP has been explored previously,^49^ we show that NT-proBNP was more predictive of heart failure, other cardiovascular diseases, and risk factors and is more influenced by genetic factors (heritability) than BNP. NT-proBNP is inactive and has a longer half-life than BNP, which likely explains the improved prediction capacity and increased heritability of plasma measures of NT-proBNP. The relationships identified with sex, ancestry, and specific cardiovascular diseases, such as atrial fibrillation, genotype-positive cardiomyopathy, and sinus bradycardia, may have implications for NT-proBNP’s predictive capacity in clinical settings (Supplemental Discussion).

#### BAG cochaperone 3 and b-type natriuretic peptides

We show that variants in *BAG3* influence plasma NT-proBNP and BNP levels (Supplemental Discussion). This is likely mediated through independent relationships with cardiomyopathy. Variants in *BAG3* have definitive evidence for causing cardiomyopathies. An example common variant (rs17617337) in the locus of *BAG3* significantly associated at GWAS with BAG3, NT-proBNP, and BNP levels (where the T-minor allele increased all three proteins). The allele has been previously associated with decreased heart failure and DCM, as well as increased ejection fraction and hypertrophy.^5,6,21,50^ This may be due to increased cell survival. It is not suggested that BAG3 and BNPs have a causal relationship; for example, the lead *NPPA/B* SNP (Figure S5; rs198379) was not significant in *BAG3* GWAS, and Mendelian randomization was not significant.

### Limitations

There are several limitations to this study. The UKB has biases (survivorship, dominated by European ancestry, etc). Assessing single-time point data limits assessments of causality and directionality. Although informative, assessments of medication use require longitudinal follow-up, and the medication associations described may be due to the diagnoses they were prescribed for. The proteomics was measured in samples collected at baseline recruitment, whereas the imaging appointment was on average 8 years later, and sensitivity analyses adjusting for this have been undertaken. Participant numbers with both proteomics and imaging are currently limited. We were limited to analyzing the circulating proteins measured in the UKB and did not select proteins involved in all aspects of disease, such as inflammation.

### Conclusions

We describe the relationships between nine plasma proteins with roles in genetic or structural cardiovascular disease or treatment pathways, which may mediate relationships between cardiovascular risk factors and disease development. We discuss the potential for additional avenues of therapeutic intervention with studies of ACE2 in hypertension and diabetes, and BAG3 in cardiomyopathies, and the need to understand the relationships with NT-proBNP for diagnostic purposes in stratified groups of patients. This study provides an improved understanding of the circulating pathways depicting cardiovascular disease dynamics and the influencing modifiers and risk factors.

## Article Information

### Sources of Funding

Dr McGurk is supported by the British Heart Foundation (BHF; FS/IPBSRF/22/27059, RE/18/4/34215). L. Curran is supported by the BHF (RE/18/4/34215). Dr Sau is supported by the BHF (FS/CRTF/21/24183) and a National Institute for Health Research (NIHR) Clinical Lectureship. Prof Ng is supported by the BHF (RG/F/22/110078, RE/18/4/34215, RE/24/130023). Dr Halliday is funded by the BHF (FS/ICRF/21/26019) and Rosetrees Trust. Prof Ware is supported by the Sir Jules Thorn Charitable Trust (21JTA), the Medical Research Council (UK) (MC_UP_1605/13), and the BHF (RG/19/6/34387, RE/18/4/34215). Prof O’Regan is supported by the Medical Research Council (MC_UP_1605/13) and the BHF (RG/19/6/34387, RE/24/130023, RG/F/24/110138, CH/F/24/90015, BBC/F/21/220106). All authors were supported by the National Institute for Health Research (NIHR) Imperial College Biomedical Research center. The views expressed in this work are those of the authors and not necessarily those of the funders. For open access, the authors have applied a CC BY public copyright license to any author-accepted manuscript version arising from this submission.

### Disclosures

Dr McGurk has consulted for Checkpoint Capital LP. Prof O’Regan has consulted for Bayer AG and Bristol Myers-Squibb. Prof Ware has consulted for MyoKardia Inc, Pfizer, Foresite Labs, Health Lumen, and Tenaya Therapeutics and has received research support from Bristol Myers-Squibb. None of these activities is directly related to the work presented here. The other authors report no conflicts.

### Supplemental Material

Supplemental Methods

Supplemental Results

Supplemental Discussion

Tables S1–S8

Figures S1–S31

References 51–60

## Supplementary Material
