# Supplementary material for: Circulating Cardiovascular Proteomic Associations With Genetics and Disease
Source: Circ Genom Precis Med. 2025 Oct 7;18(6):e005005. doi: 10.1161/CIRCGEN.124.005005 (PMC12711284; doi:10.1161/CIRCGEN.124.005005)
Supplement: Supplementary file 1 [file hcg-18-e005005-s001.docx]

# SUPPLEMENTAL MATERIAL

## Supplemental Methods

The results are expressed as mean and standard deviation (SD). The Student’s t-test was used to assess differences in means for quantitative traits and Fisher’s exact test for counts. Pearson’s correlation coefficient described relationships. Effect sizes are presented as standardised beta coefficients.

### Genetic analyses

For genome-wide association studies (GWAS), the imputed UKB genotyping data was used, where a minor allele frequency of >0.001 in autosomes was included. Individuals with more than 5% missing genotypes and SNPs with more than 5% missingness were excluded. Participant sex discrepancies, heterozygosity, and relatedness were handled by keeping only genetically European individuals and participants included in the UKB principal components analysis^18^. SNPs deviating from Hardy-Weinberg equilibrium (1x10^-8^) and those with an imputation INFO score of <0.4 were excluded.

The genotyping data for step 1 of Regenie excluded SNPs in autosomes with a minor allele frequency <0.01, missingness of >0.01, a minor allele count of <20, deviations from Hardy-Weinberg equilibrium (5x10^-15^), and individuals with greater than 10% missingness. Interchromosome, SNPs in linkage disequilibrium (indep-pairwise 1000 100 0.9), and areas of low complexity, were excluded for step 1. Exome sequencing data for step 2 was quality controlled for variants in the autosomes with missingness less than 10%, variants where less than 90% of all genotypes for that variant had a read depth less than 10, deviations from Hardy-Weinberg equilibrium (1x10^-15^), and individuals with more than 10% missingness.

### ECG analyses

Automated diagnoses had F1 scores above 80% and specificity over 99%^13^. The ECG model was previously shown to be superior cardiology resident medical doctors^13^. The ECGs were preprocessed with a bandpass filter 0.5 to 100hz, a notch filter at 60hz, and re-sampling to 400hz. Zero padding resulted in a signal with 4,096 samples for each lead for a 10s recording, which was used as input to the neural network model.

## Supplemental Results

### Medication use

39%-75% of the individuals with reported cardiovascular medications at recruitment also reported the medication at the imaging appointment: 39% on antiplatelets, 57% on beta blockers, 60% on angiotensin receptor blockers (ARBs), 65% on ACE inhibitors, 69% on calcium channel blockers, and 75% on anticoagulants.

### Cardiac ECG diagnoses and MRI parameters

The associations remained significant when individuals with reported beta-blocker use were removed from the analysis (sinus bradycardia, protein level mean difference β=0.25, P=9.44x10^-7^; atrial fibrillation, protein level mean difference β=0.83, P=0.0001). The association with atrial fibrillation was also observed for BNP (protein level mean difference β=0.81, P=2.27x10^-6^). Adjustment for the time between recruitment and imaging had little effect (NT-proBNP and sinus bradycardia, protein level mean difference β=0.27, P=2.64x10^-7^; NT-proBNP and atrial fibrillation, protein level mean difference β=1.17, P=2.29x10^-10^; BNP and atrial fibrillation, protein level mean difference β=0.96, P=1.15x10^-7^).

### NT-proBNP and BNP and their role in heart failure

Atrial fibrillation^52^ increased NT-proBNP circulating levels observationally and through Mendelian randomisation (**Figure S11, Table S8**). However, the observed association between NT-proBNP and myocardial infarction^53^ at PheWAS (**Figure 4**) and with incident myocardial infarction risk (**Figure 7, Figure S16**) did not have evidence of causality (**Table S8, Figure S12**).

## Supplemental Discussion

Six of the nine proteins assessed have been previously identified^32^ as the strongest predictors in large-scale proteomic risk scores of atrial fibrillation (NT-proBNP, BNP), cardiomyopathy (NT-proBNP, TNNI3, BNP), heart failure (NT-proBNP), hypertension (NT-proBNP, ACE2, ACTA2), nonrheumatic mitral valve disorders (NT-proBNP, BNP), pulmonary hypertension (NT-proBNP, ACE2, BNP), stable angina (NT-proBNP), hyperplasia of prostate (NT-proBNP, ACTA2, BNP), kidney disease (NT-proBNP, BNP), infections (NT-proBNP, ACTN4, BNP), and pleural effusion (NT-proBNP).

### NT-proBNP: a predictive biomarker of hypertrophic cardiomyopathy

NT-proBNP is a prohormone with an N-terminal that is cleaved to release brain or b-type natriuretic peptide 32 (BNP). BNP is released by the heart upon myocardial wall stretch; it reduces fluid and sodium retention and causes mild vasodilation, regulating blood pressure. It has been implicated in hypertrophy, fibrosis, angiogenesis, and cardiomyocyte proliferation and viability. BNP and NT-proBNP are circulating biomarkers of heart failure and hypertrophy due to the reactivation of *NPPA* and *NPPB*^54^. Here, NT-proBNP circulating levels are associated with variants in the loci of these atrial-expressed genes.

It could be suggested that the association of NT-proBNP levels with female sex may be due to lower BMI, but adjustment for BMI did not alter this finding. NT-proBNP >125 pg/mL is common in females without classical cardiovascular risk factors as well as older people^55^. The alteration in average NT-proBNP levels with ancestry is thought to convey an altered risk for hypertension^56,57^, but as average levels are lower in individuals of Chinese and African ancestry, NT-proBNP may not have similar predictive capacity across ancestries. Analyses of proteomics in more diverse ancestries would aid this assessment.

Altered NT-proBNP has been previously noted in patients with atrial fibrillation^58^, which may influence heart failure or cardiac event risk prediction capabilities in atrial fibrillation patients. Enlargement of the left atrium has been shown to increase NT-proBNP and BNP in individuals diagnosed with atrial fibrillation^59^. Enlarged atria are associated with both existing and incident atrial fibrillation and atrial fibrillation and heart failure have shared pathogenesis. The association of NT-proBNP (and BNP) with future uptake of anticoagulant medication is likely due to a diagnosis of atrial fibrillation or flutter.

The increase of NT-proBNP circulating levels in participants with a pathogenic/likely pathogenic cardiomyopathy-associated variant may be indicative of individuals at particular risk of cardiomyopathy, incident heart failure, or atrial fibrillation. The association of NT-proBNP circulating levels with sinus bradycardia has been identified previously and thought to be through increased stroke volume and wall tension^60^, and we show evidence here that it is unlikely to be due to beta-blockers prescribed for hypertension, as previously suggested^61^.

We show that variants in *BAG3* influence plasma NT-proBNP and BNP levels. We have previously described a particular common missense variant within BAG3 (C151R; rs2234962) that demonstrates BAG3’s potential cardioprotective function in GWAS of DCM, HF, and ejection fraction, alongside risk for HCM^7^. The variant is associated with proteins maintaining myofibrillar integrity and causes improved response to proteotoxic stress. BAG3’s role in the protection or risk for cardiomyopathies and carcinomas holds promise for devising therapeutic interventions, diagnostics, and tailored treatments.

## Supplemental Tables

**Table S1 Summary of participant characteristics with proteomics (n=46,011).** Mean and standard deviation or proportion (%) are presented. European ancestry was derived from genetic analyses. BMI, body mass index; SBP, systolic blood pressure. Raw protein levels are presented in Olink’s arbitrary unit in log2 scale. RVAS λGC was calculated using variant groups (M7, all).

| **Characteristic** | **mean** | **SD** | **%** | **GWAS λGC** | **RVAS λGC** |
| --- | --- | --- | --- | --- | --- |
| Age at recruitment (years) | 56.78 | 8.21 |  |  |  |
| Sex (male) |  |  | 46 |  |  |
| European ancestry (TRUE) |  |  | 93 |  |  |
| BMI (kg/m2) | 27.45 | 4.77 |  |  |  |
| SBP (mmHg) | 139.61 | 19.72 |  |  |  |
| Alcohol (g/day) | 17.50 | 21.61 |  |  |  |
| Current smoker |  |  | 11 |  |  |
| ACE2 | 0.11 | 0.64 |  | 1.04 | 1.06 |
| ACTA2 | 0.04 | 0.52 |  | 1.03 | 1.04 |
| ACTN4 | 0.02 | 0.35 |  | 1.00 | 0.99 |
| BAG3 | -0.02 | 0.46 |  | 1.04 | 1.04 |
| CDKN1A | -0.02 | 1.20 |  | 1.01 | 1.02 |
| NOTCH1 | 0.001 | 0.18 |  | 1.03 | 1.07 |
| NT-proBNP | 0.10 | 1.25 |  | 1.03 | 1.07 |
| TNNI3 | -0.08 | 0.94 |  | 1.00 | 1.02 |
| BNP | -0.02 | 1.53 |  | 1.02 | 1.05 |

**Table S2** The tables depict the self-reported medication association results for the protein traits at recruitment and at the imaging visit 8 years later. The p-value of association and beta effect size are shown. The association between proteomics at recruitment and medication reported a) at recruitment, b) at the imaging visit, c) at the imaging visit with the proteomic measures adjusted for the time from recruitment to imaging, are shown. d) lists the medications assessed by each category. N, samples with postiive medication use at baseline (46,011) or the imaging visit (5,324). NA and ns, the association with not significant. Cablocker, calcium channel blocker; anticoag, anticoagulant. (see separate file)

**Table S3** The table depicts the phenome-wide association studies results for the protein traits. SE, standard error; OR, odds ratio; p, p-value of association. (see separate file)

**Table S4** The table depicts the curated association studies results for the protein traits. The p-value of association and beta effect size are shown. NA and ns, the association with not significant. Hcm, hypertrophic cardiomyopathy; dcm, dilated cardiomyopathy; any cm, a composite of any cardiomyopathy diagnosis; muscdys, muscular dystrophy; heartfail, heart failure; musclewast, muscle wasting; repfail, respiratory failure; fibflut, atrial fibrillation and flutter; cardiacarr, cardiac arrest; valve, valve disease; hypercholesterol, hypercholesteremia. (see separate file)

**Table S5** The tables depict the relationship between MRI_derived measures and the proteins at recruitment (trait, raw measures) where the imaging visit was later in time (delta, adjusted for difference in time). The Pearson's correlation coefficient (R) is presented. FD, trabeculation; AAo, ascending aorta; DAo, descending aorta; L, left; R, right; AV, atrial volume; SV, stroke volume; EF, ejection fraction; Ell, Ecc, Err, measures of strain; EDV, end diastolic volume; ESV, end systolic volume; CO, cardiac output; M, mass; WT, wall thickness. (see separate file)

**Table S6** The table depicts the independent significant GWAS loci for the proteins. CHR, chromosome; SNP, rsid of the SNP; POS, base pair position of locus in GrCh37; A1, minor allele; A2, reference allele; N, sample size; AF1, allele frequency of the minor allele; SE, standard error; Gene, predicted gene; confirmed eQTL, whether the gene is a significant eQTL on GTEx and other potential genes from GTEx; GWAS catalog, associations previously identified from published GWAS; PheWEB, association from the PheWAS database (<https://pheweb.org/UKB-TOPMed/>). (see separate file)

**Table S7** The table depicts the protein altering variant burden analyses results for the proteins. CHROM, chromosome; GENPOS, gene position GrCh38; ALLELE1, masks; A1FREQ, allele frequency; N, sample size of discovery cohort; TEST, RVAS method analysed by Regenie software; SE, standard error; LOG10P, p-value of association. The masks are described in the Transcript column as M*; where * is M1 for LoF, M2 missense(>=1/5),missense(5/5); M3 missense(0/5),missense(>=1/5),missense(5/5); M4 missense(5/5); M6 LoF,missense(>=1/5),missense(5/5); M7 LoF,missense(0/5),missense(>=1/5),missense(5/5). all, all variants included; 0.01 or 0.001, only variants rarer than an AF of 0.01 or 0.001 were included; singletons, only singleton variants were included. (see separate file)

**Table S8** Summary of Mendelian randomisation results. The table depicts the results for proteins and dilated cardiomyopathy (DCM), hypertrophic cardiomyopathy (HCM), heart failure (HF), and systolic blood pressure. Five methods were used to assess for causality, with the number of SNPs included in the analysis (nsnp), beta effect size (b), standard error (se), and p-value (pval) shown. Tests of heterogeneity, pleiotropy, and directionality, are depicted. The mean Inverse-variance weighted F statistic and R squared of the instrumental variables are shown. (see separate file)

## Supplemental Figures

**ACE2**

**a) b)**

**
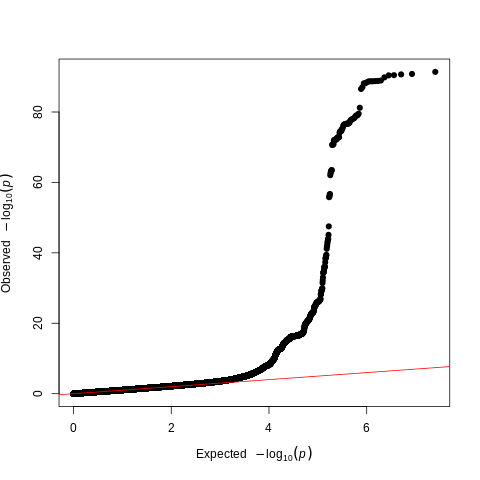

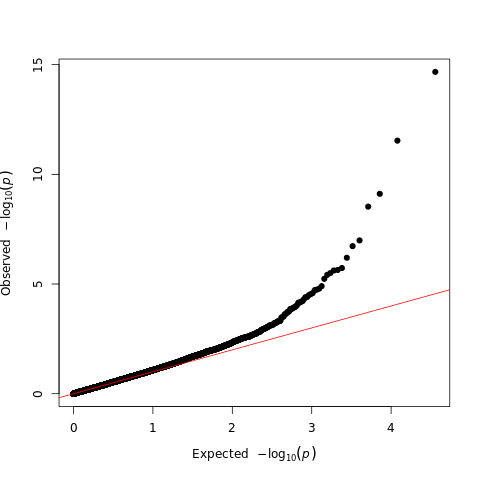
**

**ACTA2**

**a) b)**

**
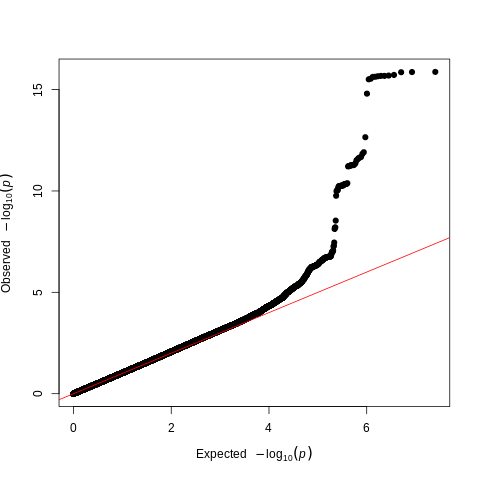

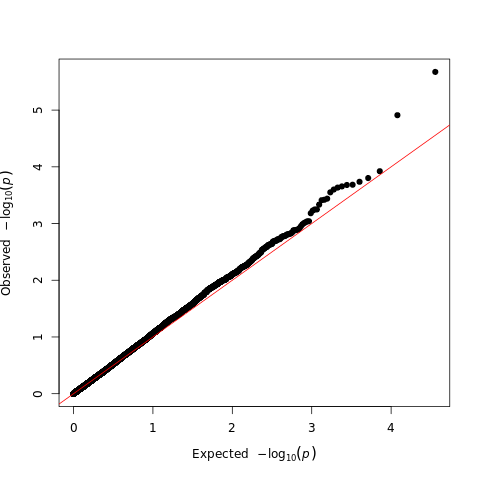
**

**ACTN4**

**a) b)**

**
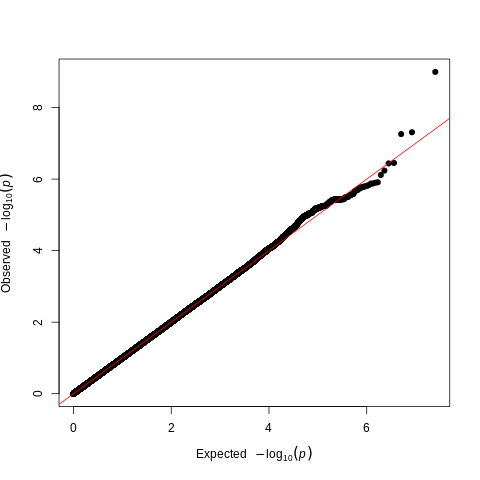

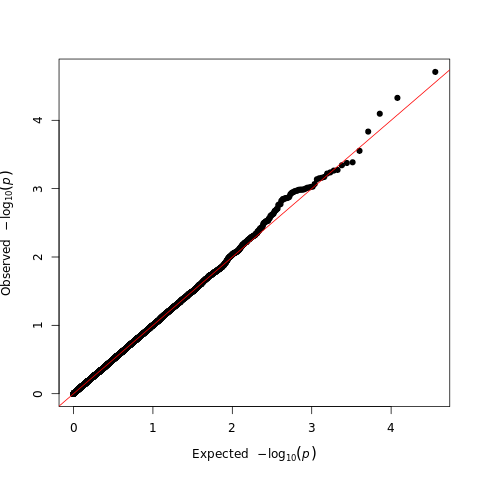
**

**BAG3**

**a) b)**

**
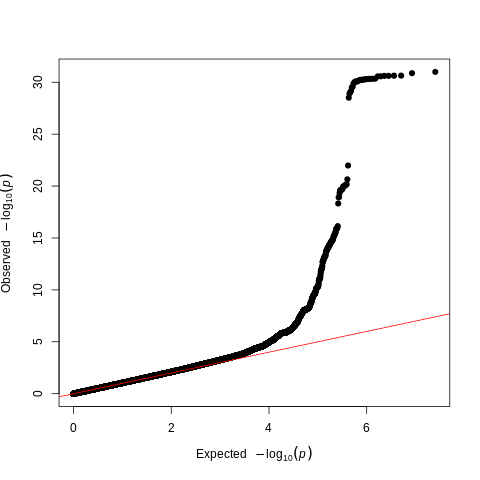

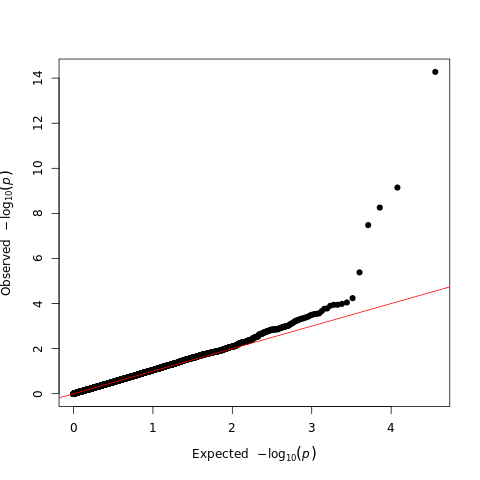
**

**BNP**

**a) b)**

**
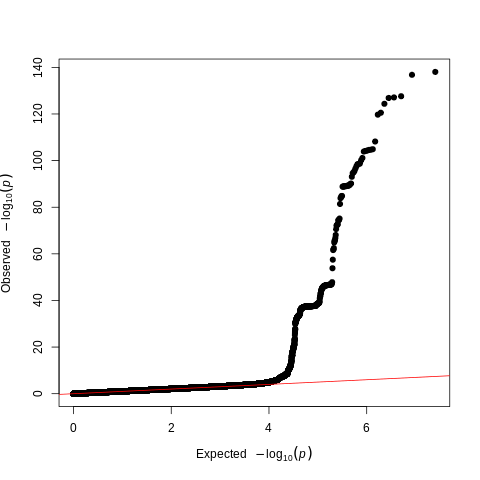

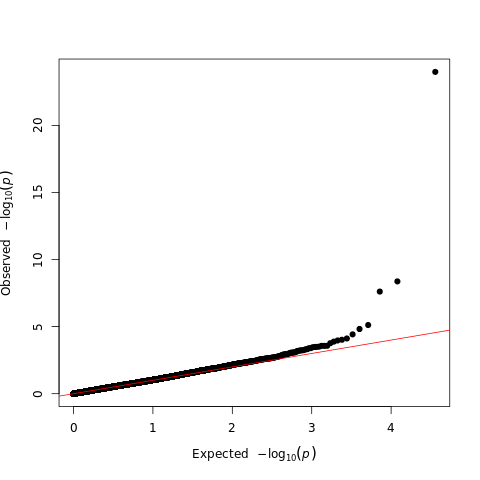
**

**CDKN1A**

**a) b)**

**
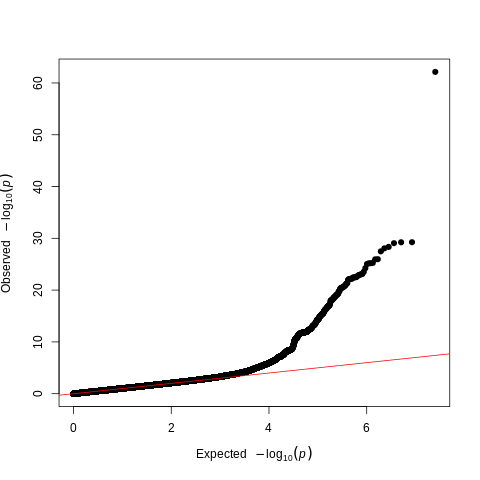

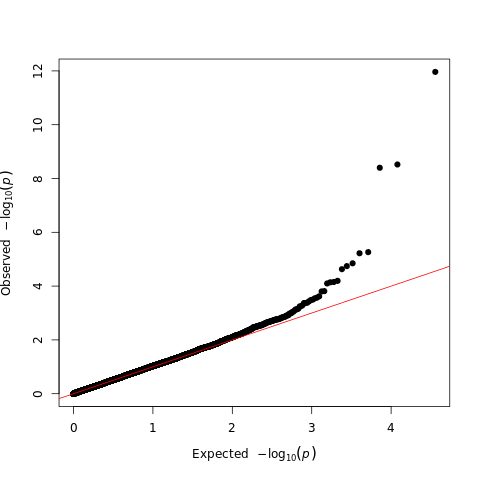
**

**NOTCH1**

**a) b)**

**
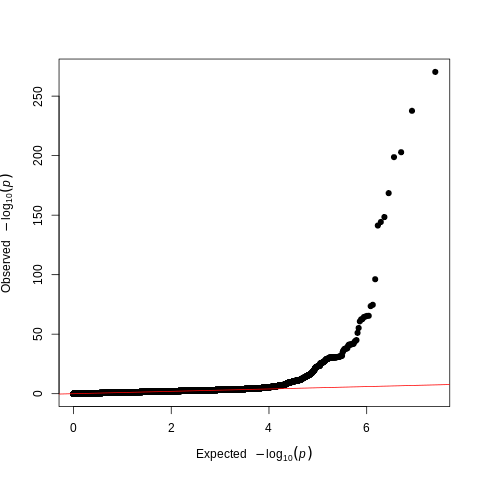

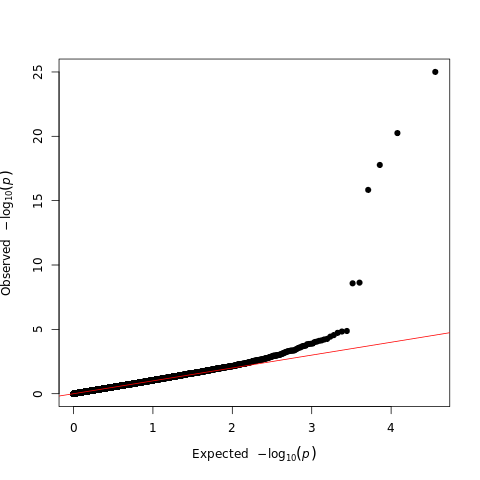
**

**NTproBNP**

**a) b)**

**
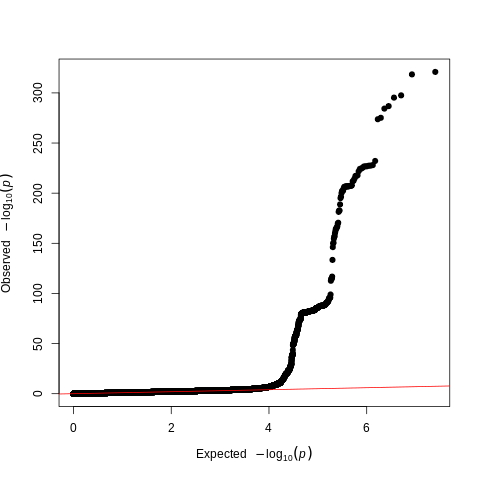

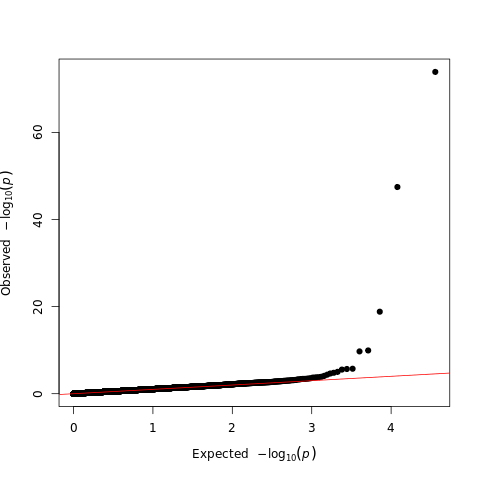
**

**TNNI3**

**a) b)**

**
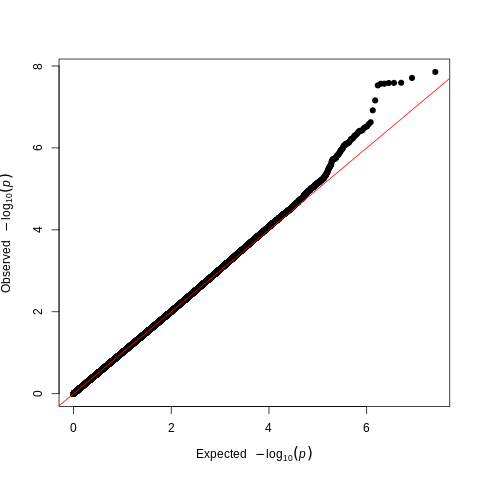

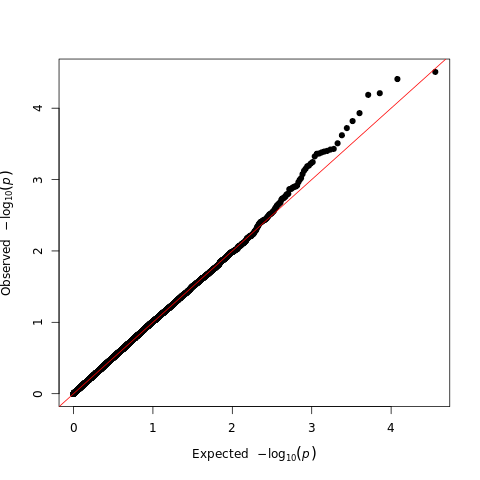
**

**Figure S1 QQ plots from a) GWAS and b) RVAS analyses.** The inflation factors can be found in **Table S1**.


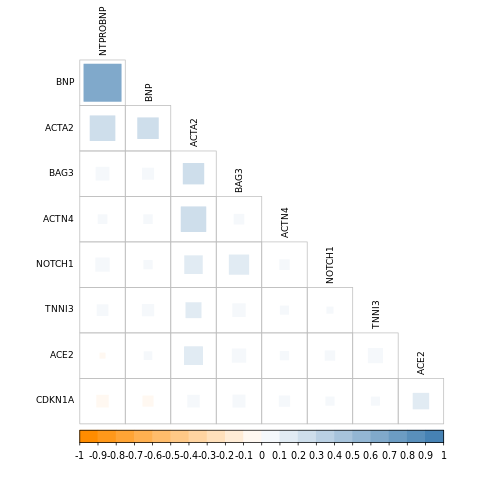


**Figure S2 Correlation between nine circulating protein levels.** The plot depicts the Pearson’s correlation coefficient (R) between the raw levels of the circulating proteins analysed. ACTA2 had the most relationships. BNP and NT-proBNP correlated with the strongest relationship to R=0.67.


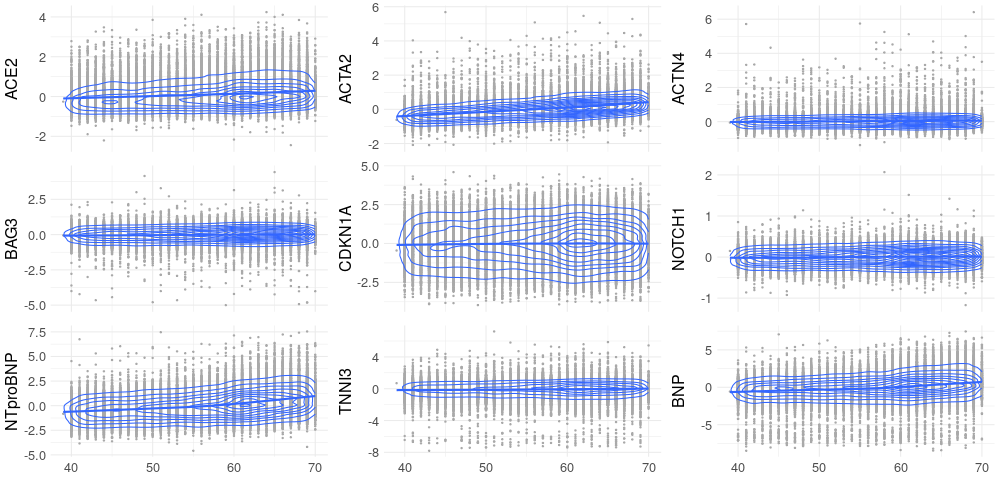


**Figure S3 The relationships with age.** The plots depict the relationships between age at recruitment (x-axis, years) and the nine plasma protein levels. Pearson’s correlation coefficient between the protein levels and age were as follows; R=0.16 ACE2, R=0.42 ACTA2, R=0.34 NT-proBNP, R=0.06 ACTN2, R=0.06 BAG3, R=0.02 CDKN1A, R=0.06 NOTCH1, R=0.06 TNNI3, and R=0.23 BNP.


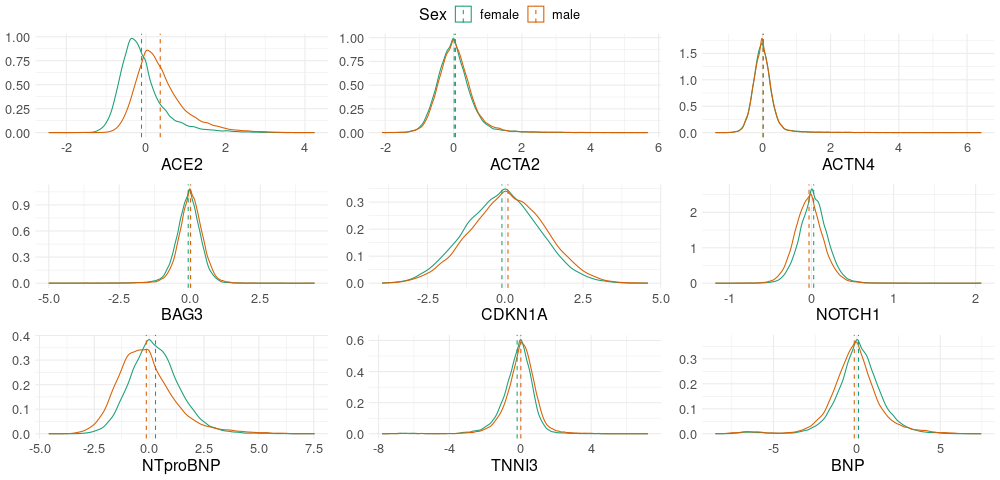


**Figure S4 The relationships with sex.** The distributions depict the relationships between sex and the nine plasma protein levels. ACE2 (P=1.0x10^-16^, β=0.47), ACTA2 (P=1.08x10^-13^, β=0.04), BAG3 (P=4.97x10^-85^, β=0.08), CDKN1A (P=6.61x10^-68^, β=0.20), and TNNI3 (P=3.97x10^-127^, β=0.21) were significantly increased with male sex. NOTCH1 (P=8.61x10^-258^, β=0.06), NT-proBNP (P=1.62x10^-278^, β=0.42), and BNP (P=3.50x10^-69^, β=0.25), were significantly increased with female sex.

**Figure S5 Relationships with medication intake adjusted for time between recruitment and imaging visit.** The plots depict the proteins measured at recruitment and adjusted for days to the imaging visit that were significantly increased with medication intake reported only at recruitment (current), or the imaging visit on average 8 years later (future), or reported during both visits (current (long-term)). The significance of differences in means as derived by Student’s t-test are denoted as stars compared to individuals with no report of the medication (never). The data only includes those with proteomics who attended the imaging visit (n=5,324).

 **Figure S6 Phenome-wide association study results of the plasma protein levels with the symptoms category of phenotypes.**

Phenotypes as phecodes are described on the y-axis and the protein traits on the x-axis. Each point denotes a significant PheWAS association with a Bonferroni correction for the number of analyzed phecodes. The shape and colour denote the direction of effect and odds ratio. See **Table S3** for the full PheWAS results. Proteins were excluded from the figures when no significant findings after correction for multiple testing were identified. Where no negative direction of association was identified, points do not have the shape of an inverted triangle.

 **Figure S7 Phenome-wide association study results of the plasma protein levels with the respiratory category of phenotypes.**

Phenotypes as phecodes are described on the y-axis and the protein traits on the x-axis. Each point denotes a significant PheWAS association with a Bonferroni correction for the number of analyzed phecodes. The shape and colour denote the direction of effect and odds ratio. See **Table S3** for the full PheWAS results. Proteins were excluded from the figures when no significant findings after correction for multiple testing were identified. Where no negative direction of association was identified, points do not have the shape of an inverted triangle.

 **Figure S8 Phenome-wide association study results of the plasma protein levels with the neurological category of phenotypes.**

Phenotypes as phecodes are described on the y-axis and the protein traits on the x-axis. Each point denotes a significant PheWAS association with a Bonferroni correction for the number of analyzed phecodes. The shape and colour denote the direction of effect and odds ratio. See **Table S3** for the full PheWAS results. Proteins were excluded from the figures when no significant findings after correction for multiple testing were identified. Where no negative direction of association was identified, points do not have the shape of an inverted triangle.

 **Figure S9 Phenome-wide association study results of the plasma protein levels with the neoplasms category of phenotypes.**

Phenotypes as phecodes are described on the y-axis and the protein traits on the x-axis. Each point denotes a significant PheWAS association with a Bonferroni correction for the number of analyzed phecodes. The shape and colour denote the direction of effect and odds ratio. See **Table S3** for the full PheWAS results. Proteins were excluded from the figures when no significant findings after correction for multiple testing were identified. Where no negative direction of association was identified, points do not have the shape of an inverted triangle.

 **Figure S10 Phenome-wide association study results of the plasma protein levels with the musculoskeletal category of phenotypes.**

Phenotypes as phecodes are described on the y-axis and the protein traits on the x-axis. Each point denotes a significant PheWAS association with a Bonferroni correction for the number of analyzed phecodes. The shape and colour denote the direction of effect and odds ratio. See **Table S3** for the full PheWAS results. Proteins were excluded from the figures when no significant findings after correction for multiple testing were identified. Where no negative direction of association was identified, points do not have the shape of an inverted triangle.

 **Figure S11 Phenome-wide association study results of the plasma protein levels with the mental disorders category of phenotypes.**

Phenotypes as phecodes are described on the y-axis and the protein traits on the x-axis. Each point denotes a significant PheWAS association with a Bonferroni correction for the number of analyzed phecodes. The shape and colour denote the direction of effect and odds ratio. See **Table S3** for the full PheWAS results. Proteins were excluded from the figures when no significant findings after correction for multiple testing were identified. Where no negative direction of association was identified, points do not have the shape of an inverted triangle.

 **Figure S12 Phenome-wide association study results of the plasma protein levels with the injuries and poisonings category of phenotypes.**

Phenotypes as phecodes are described on the y-axis and the protein traits on the x-axis. Each point denotes a significant PheWAS association with a Bonferroni correction for the number of analyzed phecodes. The shape and colour denote the direction of effect and odds ratio. See **Table S3** for the full PheWAS results. Proteins were excluded from the figures when no significant findings after correction for multiple testing were identified. Where no negative direction of association was identified, points do not have the shape of an inverted triangle.

 **Figure S13 Phenome-wide association study results of the plasma protein levels with the infectious diseases category of phenotypes.**

Phenotypes as phecodes are described on the y-axis and the protein traits on the x-axis. Each point denotes a significant PheWAS association with a Bonferroni correction for the number of analyzed phecodes. The shape and colour denote the direction of effect and odds ratio. See **Table S3** for the full PheWAS results. Proteins were excluded from the figures when no significant findings after correction for multiple testing were identified. Where no negative direction of association was identified, points do not have the shape of an inverted triangle.

 **Figure S14 Phenome-wide association study results of the plasma protein levels with the hematopoietic category of phenotypes.**

Phenotypes as phecodes are described on the y-axis and the protein traits on the x-axis. Each point denotes a significant PheWAS association with a Bonferroni correction for the number of analyzed phecodes. The shape and colour denote the direction of effect and odds ratio. See **Table S3** for the full PheWAS results. Proteins were excluded from the figures when no significant findings after correction for multiple testing were identified. Where no negative direction of association was identified, points do not have the shape of an inverted triangle.

 **Figure S15 Phenome-wide association study results of the plasma protein levels with the genitourinary category of phenotypes.**

Phenotypes as phecodes are described on the y-axis and the protein traits on the x-axis. Each point denotes a significant PheWAS association with a Bonferroni correction for the number of analyzed phecodes. The shape and colour denote the direction of effect and odds ratio. See **Table S3** for the full PheWAS results. Proteins were excluded from the figures when no significant findings after correction for multiple testing were identified. Where no negative direction of association was identified, points do not have the shape of an inverted triangle.

 **Figure S16 Phenome-wide association study results of the plasma protein levels with the endocrine and metabolic category of phenotypes.**

Phenotypes as phecodes are described on the y-axis and the protein traits on the x-axis. Each point denotes a significant PheWAS association with a Bonferroni correction for the number of analyzed phecodes. The shape and colour denote the direction of effect and odds ratio. See **Table S3** for the full PheWAS results. Proteins were excluded from the figures when no significant findings after correction for multiple testing were identified. Where no negative direction of association was identified, points do not have the shape of an inverted triangle.

 **Figure S17 Phenome-wide association study results of the plasma protein levels with the digestive category of phenotypes.**

Phenotypes as phecodes are described on the y-axis and the protein traits on the x-axis. Each point denotes a significant PheWAS association with a Bonferroni correction for the number of analyzed phecodes. The shape and colour denote the direction of effect and odds ratio. See **Table S3** for the full PheWAS results. Proteins were excluded from the figures when no significant findings after correction for multiple testing were identified. Where no negative direction of association was identified, points do not have the shape of an inverted triangle.

 **Figure S18 Phenome-wide association study results of the plasma protein levels with the dermatologic category of phenotypes.**

Phenotypes as phecodes are described on the y-axis and the protein traits on the x-axis. Each point denotes a significant PheWAS association with a Bonferroni correction for the number of analyzed phecodes. The shape and colour denote the direction of effect and odds ratio. See **Table S3** for the full PheWAS results. Proteins were excluded from the figures when no significant findings after correction for multiple testing were identified. Where no negative direction of association was identified, points do not have the shape of an inverted triangle.

 **Figure S19 Phenome-wide association study results of the plasma protein levels with the congenital anomalies category of phenotypes.**

Phenotypes as phecodes are described on the y-axis and the protein traits on the x-axis. Each point denotes a significant PheWAS association with a Bonferroni correction for the number of analyzed phecodes. The shape and colour denote the direction of effect and odds ratio. See **Table S3** for the full PheWAS results. Proteins were excluded from the figures when no significant findings after correction for multiple testing were identified. Where no negative direction of association was identified, points do not have the shape of an inverted triangle.

**a)**

**b)**

**c)**

**d)**

**Figure S20 Phenome-wide association study results of the plasma protein levels with the circulatory system category of phenotypes.**

**a)-d)** results are separated into four plots for clarity. Phenotypes as phecodes are described on the y-axis and the protein traits on the x-axis. Each point denotes a significant PheWAS association with a Bonferroni correction for the number of analyzed phecodes. The shape and colour denote the direction of effect and odds ratio. See **Table S3** for the full PheWAS results. Proteins were excluded from the figures when no significant findings after correction for multiple testing were identified. Where no negative direction of association was identified, points do not have the shape of an inverted triangle.

**a) b)**

**Figure S21 Curated cardiovascular disease association study and ACE2’s relationship with systolic blood pressure**. **a)** ICD codes as diagnoses are described on the x-axis and the protein traits are on the y-axis. Each point denotes a significant association. The shape and colour denote the direction of effect and odds ratio. The size represents the significance (where larger is more significant). See **Table S4** for the full results. DCM, dilated cardiomyopathy; HCM, hypertrophic cardiomyopathy. **b)** The linear relationship between ACE2 levels and an automatic measure of systolic blood pressure measured at recruitment (R=0.21).

**a) b)**

**c) d)**

**e) f)**

**g) h)**

**i) j)**

**k) l)**

**m) n)**

**Figure S22 Sensitivity analysis of removing all-cause death from Cox proportional hazards regression models or assessments by sex.** Deciles of ACE2 levels with incident **a)** hypertension and **b)** diabetes, since recruitment, or split by sex – **c)** hypertension in females only, **d)** hypertension in males only, **e)** diabetes in females only, **f)** diabetes in males only. Deciles of NT-proBNP levels with incident **g)** heart failure, **h)** atrial fibrillation, **i)** myocardial infarction, and **j)** cardiomyopathy, since recruitment. Deciles of BNP levels with incident **k)** heart failure, **l)** atrial fibrillation, **m)** myocardial infarction, and **n)** cardiomyopathy, since recruitment. The forest plots were created assessing diagnosis from recruitment with those diagnosed before recruitment or died without a diagnosis excluded. Sex (increasing risk is male), European ancestry (increasing risk is European), and age at recruitment, were added to this multivariable analysis for comparison.

**a) b)**

**c) d)**

**Figure S23 Mendelian randomization analysis of decreased systolic blood pressure as an exposure for ACE2 levels.** The plots show summary information on the analyses, performed as per the TwoSampleMR R package. **a)** Mendelian randomization scatter plot for decreased systolic blood pressure as an exposure for ACE2. **b)** Mendelian randomization single SNP funnel plot for decreased systolic blood pressure as an exposure for ACE2. **c)** Mendelian randomization single SNP forest plot for decreased systolic blood pressure as an exposure for ACE2. **d)** Mendelian randomization leave one out plot for decreased systolic blood pressure as an exposure for ACE2.

**a) b)**

**c) d)**

**Figure S24 Mendelian randomization analysis of ACE2 levels as an exposure for systolic blood pressure.** The plots show summary information on the analyses, performed as per the TwoSampleMR R package. **a)** Mendelian randomization scatter plot for systolic blood pressure as an outcome of ACE2 exposure. **b)** Mendelian randomization single SNP funnel plot for systolic blood pressure as an outcome of ACE2 exposure. **c)** Mendelian randomization single SNP forest plot for systolic blood pressure as an outcome of ACE2 exposure. **d)** Mendelian randomization leave one out plot for systolic blood pressure as an outcome of ACE2 exposure.**a) b)**

**c) d)**

**e) f)**

**g) h)**

**Figure S25 Mendelian randomization analysis of type-2 diabetes and ACE2 levels.** Mendelian randomisation for type-2 diabetes as an exposure **a)-d)** and outcome **e)-h)** for ACE2 levels. The plots show summary information on the analyses, performed as per the TwoSampleMR R package. **a)** Mendelian randomization scatter plot for type-2 diabetes (T2D) as an exposure for ACE2. **b)** Mendelian randomization single SNP funnel plot for T2D as an exposure for ACE2. **c)** Mendelian randomization single SNP forest plot for T2D as an exposure for ACE2. **d)** Mendelian randomization leave one out plot for T2D as an exposure for ACE2. **e)** Mendelian randomization scatter plot for ACE2 as an exposure for T2D. **f)** Mendelian randomization single SNP funnel plot for ACE2as an exposure for T2D. **g)** Mendelian randomization single SNP forest plot for ACE2as an exposure for T2D. **h)** Mendelian randomization leave one out plot for ACE2 as an exposure for T2D.

**a) b)**

**c) d)**

**e)**

**Figure S26 Increasing levels of BNP are observed in participants diagnosed with incident heart failure, cardiomyopathies, atrial fibrillation, and myocardial infarction. a)** The figure shows the sequential increase in mean BNP with a heart failure diagnosis alongside sex, age, a diagnosis of cardiomyopathy or atrial fibrillation, and carriers of pathogenic cardiomyopathy-associated variants. The Student’s t-test significance was using “No HF diagnosis” as the reference group. The groups contained the following sample sizes, respectively: (No HF diagnosis) 44050, (female groups:) 103, 286, 12, 33, 234, 2, (male groups:) 202, 496, 7, 42, 470, 14. NT-proBNP units are Olink’s arbitrary unit in log_2_ scale. HF, heart failure; plp, P/LP variant carrier; Afib, atrial fibrillation; CM, cardiomyopathy diagnosis. **b, c, d)** Forest plots of Cox proportional hazards regression models for deciles of BNP levels with incident b) heart failure, c) cardiomyopathy, d) atrial fibrillation, and e) myocardial infarction, since recruitment. The forest plots were created assessing death or diagnosis from recruitment with those diagnosed before recruitment excluded. Sex (increasing risk is male), European ancestry (increasing risk is European), and age at recruitment, were added to this multivariable analysis.

**Figure S27 NT-proBNP increases with incident cardiomyopathy.** Forest plot of cardiomyopathies by NT-proBNP deciles. The forest plots of Cox proportional hazards regression models were created assessing death or diagnosis from recruitment with those diagnosed before recruitment excluded. Sex (increasing risk is male), European ancestry (increasing risk is European), and age at recruitment, were added to this multivariable analysis.

**a) b)**

**c) d)**

**e) f)**

**g) h)**

**i) j)**

**Figure S28 Mendelian randomization analysis of cardiomyopathies as an exposure for NT-proBNP levels.** Mendelian randomisation for cardiomyopathy exposures: **a)-d)** DCM and **e)-h)** HCM, for NT-proBNP outcome. Summary plots are presented in **i)-j)**. Left ventricular hypertrophy increases NT-proBNP and BNP circulating levels, with the effect mainly through variants in *BAG3* and *CLCNKA.* The plots show summary information on the analyses, performed as per the TwoSampleMR R package. **a)** Mendelian randomization scatter plot for dilated cardiomyopathy (DCM) as an exposure for NT-proBNP. **b)** Mendelian randomization single SNP funnel plot for DCM as an exposure for NT-proBNP. **c)** Mendelian randomization single SNP forest plot for DCM an exposure for NT-proBNP. **d)** Mendelian randomization leave one out plot for DCM as an exposure for NT-proBNP. **e)** Mendelian randomization scatter plot for hypertrophic cardiomyopathy (HCM) as an exposure for NT-proBNP. **f)** Mendelian randomization single SNP funnel plot for HCM as an exposure for NT-proBNP. **g)** Mendelian randomization single SNP forest plot for HCM as an exposure for NT-proBNP. **h)** Mendelian randomization leave one out plot for HCM as an exposure for NT-proBNP. **i)** Mendelian randomization genetic determination model of CM and HF genetic instruments as exposures for NT-proBNP outcome. **j)** The effect size of the lead *NPPB* SNP (downstream variant rs198379) identified here to associate at GWAS with increased NT-proBNP and BNP levels, and from GWAS summary statistics of published case-control studies of cardiomyopathies and heart failure. The eQTL variant increases *NPPB* expression in the atria (GTEx). The results suggest that the variant increases NT-proBNP and BNP production, the risk of HCM and heart failure, and decreases the risk of DCM. See **Table S8** for further details.

**a) b)**

**c) d)**

**e) f)**

**g) h)**

**Figure S29 Mendelian randomization analysis of NT-proBNP as an exposure for cardiomyopathies.** Mendelian randomisation for cardiomyopathy outcomes: **a)-d)** DCM and **e)-h)** HCM, for NT-proBNP exposure levels. The plots show summary information on the analyses, performed as per the TwoSampleMR R package. **a)** Mendelian randomization scatter plot for NT-proBNP as an exposure for dilated cardiomyopathy (DCM). **b)** Mendelian randomization single SNP funnel plot for NT-proBNP as an exposure for DCM. **c)** Mendelian randomization single SNP forest plot for NT-proBNP as an exposure for DCM. **d)** Mendelian randomization leave one out plot for NT-proBNP as an exposure for DCM. **e)** Mendelian randomization scatter plot for NT-proBNP as an exposure for hypertrophic cardiomyopathy (HCM). **f)** Mendelian randomization single SNP funnel plot for NT-proBNP as an exposure for HCM. **g)** Mendelian randomization single SNP forest plot for NT-proBNP as an exposure for HCM. **h)** Mendelian randomization leave one out plot for NT-proBNP as an exposure for HCM.

**a) b)**

**c) d)**

**e) f)**

**g) h)**

**i)**

**Figure S30 Mendelian randomization analysis of atrial fibrillation and NT-proBNP levels.** Mendelian randomisation for atrial fibrillation (afib) as an exposure **a)-d)** and outcome **e)-h)** for NT-proBNP levels. The plots show summary information on the analyses, performed as per the TwoSampleMR R package. **a)** Mendelian randomization scatter plot for afib as an exposure for NT-proBNP. **b)** Mendelian randomization single SNP funnel plot for afib as an exposure for NT-proBNP. **c)** Mendelian randomization single SNP forest plot for afib as an exposure for NT-proBNP. **d)** Mendelian randomization leave one out plot for afib as an exposure for NT-proBNP. **e)** Mendelian randomization scatter plot for NT-proBNP as an exposure for afib. **f)** Mendelian randomization single SNP funnel plot for NT-proBNP as an exposure for afib. **g)** Mendelian randomization single SNP forest plot for NT-proBNP as an exposure for afib. **h)** Mendelian randomization leave one out plot for NT-proBNP as an exposure for afib. **i)** Summary plot of Mendelian randomization genetic determination model of atrial fibrillation genetic instruments as exposures for NT-proBNP outcome.

**a) b)**

**c) d)**

**e) f)**

**g) h)**

**Figure S31 Mendelian randomization analysis of myocardial infarction and NT-proBNP levels.** Mendelian randomisation for myocardial infarction (MI) as an exposure **a)-d)** and outcome **e)-h)** for NT-proBNP levels. The plots show summary information on the analyses, performed as per the TwoSampleMR R package. **a)** Mendelian randomization scatter plot for MI as an exposure for NT-proBNP. **b)** Mendelian randomization single SNP funnel plot for MI as an exposure for NT-proBNP. **c)** Mendelian randomization single SNP forest plot for MI as an exposure for NT-proBNP. **d)** Mendelian randomization leave one out plot for MI as an exposure for NT-proBNP. **e)** Mendelian randomization scatter plot for NT-proBNP as an exposure for MI. **f)** Mendelian randomization single SNP funnel plot for NT-proBNP as an exposure for MI. **g)** Mendelian randomization single SNP forest plot for NT-proBNP as an exposure for MI. **h)** Mendelian randomization leave one out plot for NT-proBNP as an exposure for MI.

# End
